# Supplementary material for: Additional Treatments to the Local tumour for metastatic prostate cancer-Assessment of Novel Treatment Algorithms (IP2-ATLANTA): protocol for a multicentre, phase II randomised controlled trial
Source: BMJ Open. 2021 Feb 25;11(2):e042953. doi: 10.1136/bmjopen-2020-042953 (PMC7908915; doi:10.1136/bmjopen-2020-042953)
Supplement: Supplementary data [file bmjopen-2020-042953supp001.pdf]

Supplementary Material
1.0 Assessment of progression

Baseline radiological examinations will be performed prior to enrolment. In the pilot only, prostate MRI and biopsy will be repeated at 26 (+/-12) weeks. In the main phase it is not protocol-mandated, that all patients have imaging scans repeated at the same time point (26 weeks +/- 12 weeks) and whenever clinically appropriate, such as in those with a low PSA value at enrolment or when there are concerns for progression i.e. biochemical failure, new bone pain, Skeletal Related Event (SRE). Clinicians can, at their own discretion, conduct a repeat MRI and biopsy of the prostate during the main phase.

The following outcomes should be reported:
- Biochemical failure
- Local progression
- Lymph node progression
- Bone metastases progression (new sites)
- Progression or development of new distant metastases, defined as lymph nodes outside the pelvis, bone or organ involvement
- Skeletal-related events confirmed as progression (see below).

Biochemical Failure
For the purposes of this trial, a unique threshold PSA value for biochemical failure is calculated, referred to as the PSA progression value. This value is derived for each patient based on their PSA nadir**,** defined as the lowest PSA value reported between randomisation and 6 months in the trial. The exact method for deriving the progression value for a patient depends on the value of their PSA nadir, and how this compares to their pre-treatment PSA value (i.e. the extent of the fall in PSA from the starting point).

The PSA progression value is calculated in one of three ways:
1. If the lowest recorded PSA value in the 26 weeks following randomisation is more than 4ng/ml and more than 50% of the pre-treatment PSA level then the patient fulfils the criteria for immediate treatment failure.
2. For patients whose PSA nadir in the 26 weeks following randomisation is less than or equal to 50% of the pre-treatment PSA level but remains above 4ng/ml, biochemical failure will be defined as a rise of 50% above the nadir level.
3. For patients whose PSA nadir is less than or equal to 4ng/ml, biochemical failure is defined as at least a 50% rise above the nadir value that is also above 4ng/ml.

Confirming biochemical failure: The timing and thus assessment of PSA needs to be considered because rises in PSA can occur due to non-cancer related causes such as after procedures, biopsies or urinary tract infection (UTI's). Confirmatory samples are needed in all cases of a rising PSA, prior to assigning an outcome of biochemical failure. After biochemical failure is confirmed for the first time it need not be reported again.

In the case that the raised PSA value reaches the progression value, a confirmatory PSA test should be performed after at least 1 week or 4 weeks after the completion of treatment in cases of UTI's, procedures or biopsies. Biochemical failure is confirmed

if the second value is around the same level or higher. The date of PSA progression should be provided as the date of the **first** raised PSA that fulfilled the definition for progression.

A confirmatory PSA is not required if there are other signs of progression e.g. progression of cancer related symptoms (clinical progression) or new radiological progression.

Second line treatment commenced specifically for biochemical failure should not start until the trial definition for biochemical failure has been met. However, if second line treatment does start before the trial definition is met then report the closest PSA value prior to the treatment start date as the progression value. This is not required if second line treatment is being started for other signs of progression e.g. clinical or radiological.

Testosterone levels: are required when reporting biochemical progression whilst receiving hormone treatment to confirm the diagnosis of castrate resistant prostate cancer.

Local, Lymph Node and Metastatic Failure
For each of local, lymph node and distant metastases progression, **both** the following should be reported:

- Date of first clinical/symptomatic progression
- Date of first objective/radiological progression.

Skeletal-related Events
Skeletal-related events (SREs) are defined as:

- Pathological fracture
- Spinal cord compression
- Requirement for RT to bone (e.g. for pain or impending fracture)
- Requirement for surgery (e.g. for prevention or management of fracture).

All SREs should be investigated further to establish whether or not the patient has progressed and only logged as progression if confirmed clinically or on imaging to be due to metastatic prostate cancer.

2.0 Study Management

All trial documentation, including that held at participating sites and the trial coordinating centre, will be archived for a minimum of 10 years following the end of the study. Subject files and other source data (including copies of protocols, CRFs, original reports of test results, correspondence, records of informed consent, and other documents pertaining to the conduct of the study) must be retained. Documents should be stored in such a way that they can be accessed/data retrieved later. Consideration should be given to security and environmental risks.

No study document will be destroyed without prior written agreement between the Sponsor and the investigator. Should the investigator wish to assign the study records to another party or move them to another location, written agreement must be obtained from the Sponsor.

Storage and handling of confidential trial data and documents will be in accordance with the Data Protection Act 2018 (UK).

Study Management Structure

A Trial Steering Committee (TSC) will be convened including as a minimum an Independent Chair, Independent Clinician, the Chief Investigator and Study Manager. The role of the TSC is to provide overall supervision of trial conduct and progress. Details of membership, responsibilities and frequency of meetings will be defined in a separate Charter.

A Trial Management Group (TMG) will be convened including the Chief Investigator, co-investigators and key collaborators, Study Statistician and Study Manager. The TMG will be responsible for day-to-day conduct of the trial and operational issues. Details of membership, responsibilities and frequency of meetings will be defined in separate Terms of Reference. When necessary, decisions will be referred to the TSC. Meetings will be scheduled in a risk-adapted manner to allow for the review of events during the trial.

A combined data monitoring and trial steering committee will meet twice a year basis. The composition of this committee will include but not be limited to the Chief Investigator, Trial Statistician, Trial Coordinator, Trials Unit representative, Research nurse and Patient representative

In case of early discontinuation of the study, the Follow-up Visit assessments should be performed for each subject, as far as possible.

The following reasons may result in early discontinuation:

- Early evidence that a treatment arm is harmful. If only one treatment arm is deemed to be harmful then the remaining arms of the study may continue as planned,

OR

- It is not feasible to reach the planned outcomes (A hazard ration of 0.7 to 1 will make the intervention not worth progressing with given the severe adverse effects associated with it)

The statistical criteria for termination of the study will be detailed in the statistical analysis plan (SAP).

A study-specific risk assessment will be performed prior to the start of the study to assign a risk category of 'low', 'medium' or 'high' to the trial. Risk assessment will be carried out by the ICTU QA Manager in collaboration with the Study Manager and the result will be used to guide the monitoring plan. The risk assessment will consider all aspects of the study and will be updated as required during the course of the study. The study will be monitored periodically by trial monitors to assess the progress of the study, verify adherence to the protocol, ICH GCP E6 guidelines and other national/international requirements and to review the completeness, accuracy and consistency of the data.

Monitoring procedures and requirements will be documented in a Monitoring Plan, developed in accordance with the risk assessment.

Quality Control will be performed according to ICTU/ internal procedures. The study may be audited by a Quality Assurance representative of the Sponsor and/or ICTU. All necessary data and documents will be made available for inspection.

The study may be subject to inspection and audit by regulatory bodies to ensure adherence to GCP and the NHS Research Governance Framework for Health and Social Care (2nd Edition).

Dissemination of findings

Information concerning the study, patent applications, processes, scientific data or other pertinent information is confidential and remains the property of the Sponsor. The investigator may use this information for the purposes of the study only.

It is understood by the investigator that the Sponsor will use information developed in this clinical study in connection with the development of the ablative or radiotherapy or surgical techniques and, therefore, may disclose it as required to other clinical investigators and to Regulatory Authorities. In order to allow the use of the information derived from this clinical study, the investigator understands that he/she has an obligation to provide complete test results and all data developed during this study to the Sponsor.

Verbal or written discussion of results prior to study completion and full reporting should only be undertaken with written consent from the Sponsor.

Therefore all information obtained as a result of the study will be regarded as CONFIDENTIAL, at least until appropriate analysis and review by the investigator(s) are completed.

Permission from the Executive/Writing Committee is necessary prior to disclosing any information relative to this study outside of the Trial Steering Committee. Any request by site investigators or other collaborators to access the study dataset must be formally reviewed by the TMG.

The results may be published or presented by the investigator(s), but the Funder will be given the opportunity to review and comment on any such results for up to 1 month before any presentations or publications are produced.

A Clinical Study Report summarising the study results will be prepared and submitted to the REC within a year of the end of study.

3.0 Informed Consent Form

ATLANTA

Additional **T**reatments to the **L**ocal tumour for metastatic prostate cancer: **A**ssessment of **N**ovel **T**reatment **A**lgorithms

INFORMED CONSENT FORM

Chief Investigator: Professor Hashim U. Ahmed

Principal Investigator: <<Insert Principal Investigator>>

Please initial each box below. Do not tick

1	I confirm that I have read and understand the patient information sheet dated _ _ / _ _ / _ _ _ _ (Version ___) for the **ATLANTA** Study. I have had the opportunity to consider the information, ask questions and have had these answered satisfactorily.	
2	I understand that the type of treatment I receive will be allocated using a randomisation process, and neither myself nor the staff involved in the study can influence this allocation.	
3	I understand that if at any point my medical condition changes, it may be necessary to withdraw from the trial and have treatment options reviewed. This will be discussed with me by clinicians and with my agreement.	
4	I understand that I may be asked questions relating to personal aspects such as about diet and lifestyle from my local research team.	
5	I understand that my participation is voluntary and that I am free to withdraw at any time, without giving reason and without my medical care or legal rights being affected.	
6	I understand that relevant sections of my medical notes and data collected during the study, may be looked at by individuals from the Sponsor of the trial (Imperial College London) and responsible persons authorised by the Sponsor, from regulatory authorities or from the NHS Trust, where it is relevant to my taking part in this research. I give permission for these individuals to have access to my records.	
7	I understand that the information collected about me will be used to support other research in future, and may be shared anonymously with other researchers.	
8	I give permission for all standard of care data and samples, including those taken prior to study recruitment, such as surgery specimen and biopsies including tissue, bloods, urine and imaging tests to be used in this study even if I withdraw at any points from the study	

9	I give permission for the researchers to contact me regarding this trial during this trial period.	
10	I agree for my GP and other doctors to be informed of my participation in this study and of any clinical relevant study results	
11	I agree to take part in the above study.	

All the boxes above must be initialled for consent to be valid

Please initial each box below. Do not tick

OPTIONAL		
12	I give permission for additional blood and urine samples to be taken and be made available for future research where the samples would be stored appropriately and the research approved separately ***(If you do not wish to give this permission, do not initial – you can still participate in the study).***	
13	I give permission for all standard of care samples and data such as surgery specimen, biopsies including tissue as well as imaging scans to be made available for future research where the samples and scans would be stored appropriately and the research approved separately ***(If you do not wish to give this permission, do not initial – you can still participate in the study).***	
14	I give permission for any blood, urine and tissue samples, which will look for changes in my genetic material (DNA) as described in the information sheet, to be used in this study. ***(If you do not wish to give this permission, do not initial – you can still participate in the study).***	
15	I give permission for any blood, urine and tissue samples, which will look for changes in my genetic material (DNA) as described in the information sheet, to be used for further ethically approved research in the field of prostate cancer research. ***(If you do not wish to give this permission, do not initial – you can still participate in the study).***	
16	I give permission for my samples and data from any scans to be sent and utilised in research both in the UK and worldwide. All material will be anonymous and I will not be identifiable. I understand that I will not be asked again for permission to run these additional research tests and I may also not be informed of the results ***(If you do not wish to give this permission, do not initial – you can still participate in the study).***	
17	I give permission for the researchers to contact me in the future regarding the possibility of further studies, but I understand that I am under no obligation to take part in these ***(If you do not wish to give this permission, do not initial – you can still participate in the study).***	
18	I give permission for all samples taken to be biobanked and transferred to the Imperial College Healthcare Tissue Bank (ICHTB) or other UK-based biobank for a period of up to 10 years (or per local policy) and will be used for histological, genomic and epigenetic analysis and for ethically approved future	

	studies by our team or other scientists interested in prostate cancer research. Samples will not have any personal information written on them. Researchers will not be able to identify you from your samples. ***(If you do not wish to give this permission, do not initial – you can still participate in the study).***	
19	I give permission for my name and NHS number to be used to obtain information about my health status from records held by the NHS and maintained by the NHS Information Centre and the NHS Central Register or any applicable NHS information system (including linkage to routine hospital admission data). I give this consent solely so that researchers may follow up on my health status for 10 years after my participation in the study. ***(If you do not wish to give this permission, do not initial – you can still participate in the study).***	
20	I give permission for my partial postcode to be recorded and stored. This will be used for research purposes only and will remain confidential. ***(If you do not wish to give this permission, do not initial – you can still participate in the study***).	

Name of Participant Signature Date

____________________________ _____________________ _______________

Name of Person taking consent Signature Date

____________________________ ____________________ ________________

Please give one copy of the consent form to the patient, file one copy in the patient's medical records, and retain the original in the Investigator Site File
