# Supplementary material for: Additional Treatments to the Local tumour for metastatic prostate cancer-Assessment of Novel Treatment Algorithms (IP2-ATLANTA): protocol for a multicentre, phase II randomised controlled trial
Source: BMJ Open. 2021 Feb 25;11(2):e042953. doi: 10.1136/bmjopen-2020-042953 (PMC7908915; doi:10.1136/bmjopen-2020-042953)
Supplement: Author's manuscript [file bmjopen-2020-042953.draft_revisions.pdf]

BMJ Open is committed to open peer review. As part of this commitment we make the peer review history of every article we publish publicly available.

When an article is published we post the peer reviewers' comments and the authors' responses online. We also post the versions of the paper that were used during peer review. These are the versions that the peer review comments apply to.

The versions of the paper that follow are the versions that were submitted during the peer review process. They are not the versions of record or the final published versions. They should not be cited or distributed as the published version of this manuscript.

BMJ Open is an open access journal and the full, final, typeset and author-corrected version of record of the manuscript is available on our site with no access controls, subscription charges or pay-per-view fees (http://bmjopen.bmj.com).

If you have any questions on BMJ Open's open peer review process please email info.bmjopen@bmj.com

BMJ Open

Additional Treatments to the Local tumour for metastatic prostate cancer: Assessment of Novel Treatment Algorithms, a multicentre, phase II randomised controlled trial (IP2-ATLANTA)

Journal:	*BMJ Open*
Manuscript ID	bmjopen-2020-042953
Article Type:	Protocol
Date Submitted by the Author:	24-Jul-2020

Complete List of Authors:	Connor, Martin; Imperial College London, Department of Surgery and Cancer; Imperial College Healthcare NHS Trust, Imperial Urology<br>Shah, Taimur T; Imperial College London, Department of Surgery and Cancer<br>Smigielska, Katarzyna; Imperial College London, Department of Surgery and Cancer; Imperial College London, Imperial College Clinical Trials Unit<br>Day, Emily; Imperial College London, Imperial College Clinical Trials Unit<br>Sukumar, Johanna; Imperial College London, Department of Surgery and Cancer; Imperial College London, Imperial College Clinical Trials Unit<br>Fiorentino, Francesca; Imperial College London, Imperial College Clinical Trials Unit<br>Sarwar, Naveed; Imperial College Healthcare NHS Trust, Department of Oncology<br>Gonzalez, Michael; Imperial College Healthcare NHS Trust, Department of Oncology<br>Falconer, Alison; Imperial College Healthcare NHS Trust, Department of Oncology<br>Klimowska-Nassar, Natalia; Imperial College London, Department of Surgery and Cancer; Imperial College London, Imperial College Clinical Trials Unit<br>Evans , Martin; Imperial College London, Department of Surgery and Cancer<br>Naismith, Olivia; Royal Marsden NHS Foundation Trust, Radiotherapy Trials Quality Assurance (RTTQA)<br>Thippu Jayaprakash, Kamalram; Addenbrooke's Hospital, Department of Oncology<br>Price, Derek; Imperial College London, Department of Surgery and Cancer<br>Gayadeen, Shiva; Imperial College Healthcare NHS Trust, Department of Oncology<br>Basak, Dolan; Imperial College Healthcare NHS Trust, Department of Oncology<br>Horan, Gail; Addenbrooke's Hospital, Department of Oncology<br>McGrath, John; Royal Devon and Exeter NHS Foundation Trust, Urology<br>Sheehan, Denise; Royal Devon and Exeter NHS Foundation Trust, Department of Oncology<br>Kumar, Manal; Arrowe Park Hospital, Department of Urology<br>Ibrahim , Azman; Clatterbridge Cancer Centre NHS Foundation Trust,

	Department of Clinical Oncology<br>Brock, Cathryn; Chelsea and Westminster Hospital NHS Foundation Trust, Department of Oncology<br>Pearson, Rachel; Newcastle Upon Tyne Hospitals NHS Foundation Trust, Department of Oncology<br>Anyamene , Nicola; London North West University Healthcare NHS Trust, Department of Oncology<br>Heath , Catherine; University Hospital Southampton NHS Foundation Trust, Department of Radiotherapy<br>Shergill, Iqbal; Wrexham Maelor Hospital, Department of Urology<br>Rai, Bhavan; Newcastle Upon Tyne Hospitals NHS Foundation Trust, Department of Urology<br>Hellawell, Giles; London North West University Healthcare NHS Trust, Department of Urology<br>Mccracken, Stuart; Sunderland Royal Hospital, Department of Urology<br>Khoubehi, Bijan; Chelsea and Westminster Healthcare NHS Trust, Department of Urology<br>Mangar, Stephen; Imperial College Healthcare NHS Trust, Department of Oncology<br>Khoo, Vincent; Royal Marsden NHS Foundation Trust, Department of Oncology<br>Dudderidge, Tim; University Hospital Southampton NHS Foundation Trust, Department of Urology<br>Staffurth, John; Velindre Cancer Centre, Research; Cardiff University School of Medicine, Division of Cancer and Genetics<br>Winkler, Mathias; Imperial College London, Department of Surgery and Cancer; Imperial College Healthcare NHS Trust, Imperial Urology<br>Ahmed, Hashim; Imperial College London Division of Surgery, Imperial College London
Keywords:	Prostate disease < UROLOGY, Radiation oncology < RADIOTHERAPY, RADIOTHERAPY, UROLOGY, Urological tumours < UROLOGY, Genitourinary imaging < RADIOLOGY & IMAGING

SCHOLARONE™
Manuscripts

I, the Submitting Author has the right to grant and does grant on behalf of all authors of the Work (as defined in the below author licence), an exclusive licence and/or a non-exclusive licence for contributions from authors who are: i) UK Crown employees; ii) where BMJ has agreed a CC-BY licence shall apply, and/or iii) in accordance with the terms applicable for US Federal Government officers or employees acting as part of their official duties; on a worldwide, perpetual, irrevocable, royalty-free basis to BMJ Publishing Group Ltd ("BMJ") its licensees and where the relevant Journal is co-owned by BMJ to the co-owners of the Journal, to publish the Work in this journal and any other BMJ products and to exploit all rights, as set out in our licence.

The Submitting Author accepts and understands that any supply made under these terms is made by BMJ to the Submitting Author unless you are acting as an employee on behalf of your employer or a postgraduate student of an affiliated institution which is paying any applicable article publishing charge ("APC") for Open Access articles. Where the Submitting Author wishes to make the Work available on an Open Access basis (and intends to pay the relevant APC), the terms of reuse of such Open Access shall be governed by a Creative Commons licence – details of these licences and which Creative Commons licence will apply to this Work are set out in our licence referred to above.

Other than as permitted in any relevant BMJ Author's Self Archiving Policies, I confirm this Work has not been accepted for publication elsewhere, is not being considered for publication elsewhere and does not duplicate material already published. I confirm all authors consent to publication of this Work and authorise the granting of this licence.

For peer review only

Additional Treatments to the Local tumour for metastatic prostate cancer: Assessment of Novel Treatment Algorithms, a multicentre, phase II randomised controlled trial (IP2-ATLANTA)

Martin J. Connor [1,2,3], Taimur T Shah [1], Kasia Smigielska [1,4], Emily Day [1,4], Johanna Sukumar [1, 4], Francesca Fiorentino [1,4], Naveed Sarwar [5], Michael Gonzalez [5], Alison Falconer [5], Natalia Klimowska-Nassar [1, 4], Martin Evans [1, 4], Olivia Frances Naismith [6], Kamalram Thippu Jayaprakash [7], Derek Price [1], Shiva Gayadeen [5], Dolan Basak [5], Gail Horan [7], John McGrath [8], Denise Sheehan [8], Manal Kumar [9], Azman Ibrahim [10], Cathryn Brock [11], Rachel A. Pearson [12], Nicola Anyamene [13], Catherine Heath [14], Iqbal Shergill [15], Bhavan Rai [12], Giles Hellawell [13], Stewart McCracken [16], Bijan Khoubehi [11], Stephen Mangar [5], Vincent Khoo [17], Tim Dudderidge [14], John Nicholas Staffurth [18], Mathias Winkler [1,2,3], Hashim U. Ahmed [1,2]

1. Division of Surgery, Imperial Prostate, Department of Surgery and Cancer, Faculty of Medicine, Imperial College London, London, UB1 3HW
2. Imperial Urology, Imperial College Healthcare NHS Trust, London, W6 8RF
3. Department of Urology, West Middlesex University Hospital (WMUH), Chelsea & Westminster NHS Foundation Trust, Isleworth, Middlesex, TW7 6AF
4. Imperial Clinical Trials Unit (ICTU), School of Public Health, Faculty of Medicine, Imperial College London, London, UB1 3HW
5. Department of Oncology, Imperial College Healthcare NHS Foundation Trust, Charing Cross Hospital, London, W6 8RF
6. Radiotherapy Trials Quality Assurance (RTTQA), Department of Physics, The Royal Marsden NHS Foundation Trust, London, SW3 6JJf
7. Department of Oncology, Addenbrooke's Hospital and The Queen Elizabeth Hospital Kings Lynn NHS Foundation, Norfolk, PE30 4ET
8. Department of Urology & Oncology, Royal Devon & Exeter Hospital, Exeter EX2 5DW

For peer review only - http://bmjopen.bmj.com/site/about/guidelines.xhtml

9. Department of Urology, Arrowe Park Hospital, Wirral University NHS Foundation Trust, Merseyside, CH49 5PE
10. Department of Oncology, Clatterbridge Cancer Centre NHS Foundation Trust, Wirral, CH63 4JY
11. Department of Urology & Oncology, Chelsea and Westminster Hospital, Chelsea & Westminster NHS Foundation Trust, SW10 9NH.
12. Department of Urology & Oncology, Northern Centre for Cancer Care, Newcastle upon Tyne Hospitals NHS Foundation Trust, NE7 7DN
13. Department of Urology & Oncology, Northwick Park Hospital, London Northwest University Healthcare NHS Trust, Harrow, HA1 3UJ
14. Department of Urology & Oncology, University Hospital Southampton NHS Foundation Trust, Southampton, SO16 6YD
15. Department of Urology, Wrexham Maelor Hospital, Wales, LL13 7TD
16. Department of Urology, Sunderland Royal Hospital, Sunderland, SR4 7TP
17. Department of Clinical Oncology, The Royal Marsden Hospital & Institute of Cancer Research, London, SW3 6JJ,
18. School of Medicine, Cardiff University, Cardiff, Wales, CF10 3AT

Corresponding Author:

Mr. Martin J. Connor

Division of Surgery

Imperial Prostate

Department of Surgery & Cancer

Imperial College London

Room 5L.28

Charing Cross Lab Block

m.connor@imperial.ac.uk

Keywords: *prostate cancer, metastatic, radiotherapy, radical prostatectomy, cytoreductive, SABR, stereotactic radiotherapy, high intensity focussed ultrasound, cryotherapy, oligo-metastatic disease, metastasis directed therapy.*

For peer review only

Abstract

Introduction: Survival in men diagnosed with *de novo* synchronous metastatic prostate cancer has increased following the use of upfront systemic treatment, using chemotherapy and other novel androgen receptor targeted agents, in addition to standard androgen deprivation therapy (ADT). Local cytoreductive and metastasis-directed interventions are hypothesised to confer additional survival benefit. In this setting, IP2-ATLANTA will explore progression-free survival (PFS) outcomes with the addition of sequential multi-modal local and metastasis-directed treatments compared to standard care alone.

Methods: A phase II, prospective, multi-centre, three-arm randomised controlled trial incorporating an embedded feasibility pilot. All men with new histologically diagnosed, hormone-sensitive, metastatic prostate cancer, within four months of commencing ADT and of performance status 0 to 2 are eligible. Patients will be randomised to: Control (Standard of Care [SOC]) OR Intervention 1 (minimally invasive ablative therapy to prostate +/- pelvic lymph node dissection [PLND]) OR Intervention 2 (cytoreductive radical prostatectomy +/- PLND OR prostate radiotherapy +/- pelvic lymph node radiotherapy [PLNRT]). Metastatic burden will be pre-specified using the CHAARTED definition. Men with low burden disease in intervention arms are eligible for metastasis-directed therapy, in the form of stereotactic ablative body radiotherapy (SABR) or surgery. Standard systemic therapy will be administered in all arms With ADT +/- upfront systemic chemotherapy or androgen receptor agents. Patients will be followed-up for a minimum of two-years. Primary outcome: PFS. Secondary outcomes include: predictive factors for PFS and overall survival; urinary, sexual and rectal side-effects. Embedded feasibility sample size is 80, with 918 patients required in the main phase II component.

Ethics and dissemination: Approved by HRA Wales REC 5 (19/WA0005). Funded by the Wellcome Trust (204998/Z/16/Z).

Registration details: NCT03763253; ISCRTN58401737

For peer review only - http://bmjopen.bmj.com/site/about/guidelines.xhtml

STRENGTHS AND LIMITATIONS OF THIS STUDY

1. IP2-ATLANTA addresses an important research gap in the role of local and metastasis-directed therapy in men with newly-diagnosed metastatic prostate cancer.
2. This is the first phase II trial to include cytoreductive minimally invasive ablative therapy alongside cytoreductive radical prostatectomy and prostate radiotherapy.
3. The IP2-ATLANTA study builds on the clinical benefits derived from metastasis-directed therapy (stereotactic ablative body radiotherapy and/or surgery) in a previously untreated cohort of men with advanced disease.
4. Due to invasive interventions, blinding is not possible in the IP2-ATLANTA study.

For peer review only

INTRODUCTION

Overall, 47,000 men are diagnosed with prostate cancer each year in the United Kingdom (UK) [1]. Approximately, 4,500 of these men this will be diagnosed with *de novo* synchronous metastatic disease at presentation [1]. As with the United States, where just under 8% present with metastatic disease and where the annual burden is predicted to reach approximately 15,000 cases by 2025, so the prediction for the same magnitude is likely for the UK [2].

Traditionally, such men were managed with androgen deprivation therapy (ADT) alone, via medical or surgical castration [2]. Unfortunately the median time to the emergence of a castrate resistant state is in the order of 11 to 18 months, limiting overall survival (OS) to 3.5 years [3, 4]. Promisingly, the reported OS in this group has now risen to a median 4.8 years with the addition of upfront systemic agents, such as Docetaxel, Enzalutamide, Abiraterone Acetate or Apalutamide [5-9].

Moving beyond early systemic therapy escalation there has been an increased focus on the role of local cytoreductive and metastasis-directed interventions (primarily stereotactic ablative body radiotherapy [SABR]) to gain additional oncological benefit [2]. This is in part based upon the emergence of the 'oligo-metastatic state', which may exhibit different biological characteristics to poly-metastatic prostate cancer [10].

Such men present with a clinically-defined favourable metastatic burden, and are hypothesised to occupy an intermediate state between 'locally advanced' and 'poly-metastatic' disease [10, 11]. It is postulated, but not proven, that men exhibiting such disease patterns gain most benefit in progression-free survival (PFS) and OS resulting from localised cancer control achieved via cytoreductive interventions [2, 10].

PATHOBIOLOGICAL BASIS FOR LOCAL CYTOREDUCTIVE & METASTASIS DIRECTED THERAPY

The pathobiological basis underpinning local cytoreductive and metastasis-directed therapy in prostate cancer is not fully delineated. Indeed, numerous theories exist surrounding the role of the primary tumour and its relationship with metastases [2].

For peer review only - http://bmjopen.bmj.com/site/about/guidelines.xhtml

For peer review only

Local prostate cytoreduction is thought to primarily impact on tumour-derived factors such as cytokines, chemokines and microRNAs [12, 13]. In particular, prostate tumour cell shedding and dissemination has been shown to occur earlier, with the detection of circulating tumour cells (CTCs) in blood and disseminated tumour cells (DTCs) in bone marrow of patients staged as non-metastatic on conventional imaging (i.e. bone scintigraphy) [14-18].

This has led to a comparison to the “self-seeding” hypothesis, as described in other solid organ malignancies involving the breast and colon [19, 20]. It posits that the return of CTCs or DTCs from distant secondary sites alters the primary tumour microenvironment (TME), via release of matrix metalloproteinases (e.g. matrix metalloproteinase-1) and cytokines (e.g. CXC-motif chemokine 1) [19, 20] . In addition, the primary tumour cells may also secrete interleukins (e.g. IL-6) that attract further CTCs and promote local tumour angiogenesis [20, 21].

Such circulation may lead not only to “self-seeding” but also the remodelling of a “pre-metastatic niche” at new distant sites [19, 22, 23]. Bone marrow-derived haematopoietic cells localise to support pre-metastatic niche’s, promoting the local environment for colonisation [14, 16]. Bone marrow-derived endothelial and mesenchymal cells are implicated in the maturation of micro- to macro-metastasis [23]. Pre-clinical models have reported these cells are mobilised largely as a results of secretion of vascular endothelial growth factor (VEGF) and placental growth factor (PIGF) by the primary tumour [23-25].

Furthermore, investigators utilising multifocal sequencing approaches have revealed the present of primary-tumour-to-metastasis, but also surprisingly, metastasis-to-metastasis transfer of clonal tumour cells [26]. This subsequently lead to the exploration of metastasis-directed therapy [27, 28].

Metastasis-directed therapy commonly takes the form of ionizing radiation (i.e. SABR). Such interventions are hypothesised to have an effect on distant tumours via the release of tumour antigens, damage-associated molecular patterns (DAMPs), and local activation of immune cells (including cytotoxic T-cells) [29-32]. This has been coined the ‘abscopal effect’ and is associated with the generation of a systemic anti-

For peer review only - http://bmjopen.bmj.com/site/about/guidelines.xhtml

tumour immune response. Evidence for such a response in prostate cancer remains sparse [27, 33].

However, the recent ORIOLE phase II randomised study evaluated metastasis-directed SABR, versus observation alone, in 54 men with metachronous disease. At 6-months progression occurred in 7/36 (19%) men treated with SABR, compared to 11/18 (61%) in the observation arm. Interestingly, the study reported clusters of similar expanded T-cell receptors in some men (*n* = 3) in the treatment arm alone [27].

Immune-mediate responses may not be limited to cytotoxic radiotherapy, with minimally invasive ablative therapy (MIAT), such as the 'cryo-immunological response' induced by cryotherapy, also described [34]. Such ablation leads to tumour-associated antigens (TAAs), this may result in an anti-tumour response (humoral or cellular) [35]. Activation of TAA-specific T-cells may occur at time of tissue necrosis [34].

Similar to the cytotoxic abscopal response, the clinical translation of such observed responses is unclear [2]. Early local prostate cryotherapy case series reported spontaneous distant regression of metastasis, although this has not been replicated in the contemporary literature [34, 36]. Furthermore, clinical augmentation of prostate cryotherapy by immune-checkpoint inhibitors (e.g. anti-programmed cell death-1 antibody, PD-1) also demonstrated pre-clinical promise, but proved disappointing when translated into early phase clinical studies [37, 38]. Generally considered a low-immunogenic disease, it is likely that if any oncological benefit is to be derived, it will be reported when immunotherapy is added to existing proven systemic agents [39].

When taken collectively, removal of the primary tumour and possibly its metastatic sites, may lead to a disruption in these immune-mediated pathological relationships and result in regression of metastases with a prolonged cancer-specific survival (CSS).

CATEGORISING METASTATIC BURDEN

A key research barrier at present is that there is no universally accepted definition for oligometastatic disease, which varies depending on the anatomical site (nodal, burden, visceral), absolute number (1 to 7), temporal pattern (outside vertebral bodies

For peer review only - http://bmjopen.bmj.com/site/about/guidelines.xhtml

or pelvis) and diagnostic imaging utilised (conventional or molecular) [11]. Consequently there is also no accepted definition for ‘high’ versus ‘low’ volume disease [10, 40, 41]. At present oncology trials exploring systemic therapy have frequently adopted the use of the CHAARTED definition of metastatic disease burden. High burden disease is defined as four or more bone metastases with one or more metastasis located outside the vertebral bodies or pelvis, and/or visceral metastasis present [9, 42].

CYTOREDUCTIVE PROSTATE RADIOTHERAPY

Two randomised studies (STAMPEDE and HORRAD) have evaluated the role of cytoreductive local prostate radiotherapy in this cohort [42, 43].

The Systemic Therapy in Advancing Or Metastatic Prostate Cancer: Evaluation Of Drug Efficacy (STAMPEDE) collaborators explored the role of local prostate radiotherapy in 2,061 men with newly diagnosed metastatic prostate cancer receiving ADT, with 18% also receiving Docetaxel. Although no OS advantage was demonstrated (HR 0.92, [95% CI 0.80-1.06]; *p*=0.27) in all burden metastatic disease, radiotherapy did improve failure-free survival (HR 0·76, [95% CI 0·68–0·84]; *p*<0·0001) [42]. Nevertheless, in the pre-specified subgroup of men with the CHAARTED definition of low burden disease, a significant OS was reported (3-year OS 81% vs 73%, HR 0.68, [95% CI 0.52–0.90]; *p*=0.007). As with any sub-group analyses this data needs it be interpreted cautiously. Furthermore, radiotherapy treatment (weekly or daily) had acceptable side-effects with only a 5% grade 3-4 adverse event rate [42].

The HORRAD phase three trial randomised 432 men to ADT with or without local prostate radiotherapy. In accordance with STAMPEDE, no significant difference in OS was observed between the two groups (median 45 months in experimental arm versus 43 months with ADT alone; HR 0.90, [95% CI 0.70–1.14]; *p* = 0.40), though there was a non-significant trend towards improved overall survival in the 160 patients with low volume metastatic disease treated with radiotherapy (HR 0.68, 95% CI 0.42-1.10). This study however was criticised for its lack of pre-specified metastatic burden (including no knowledge of visceral disease) and potential underpowered sample size [44]. Both trials took place at a time when upfront systemic agents had not been fully

For peer review only - http://bmjopen.bmj.com/site/about/guidelines.xhtml

introduced and thus the true “additive” effect of local prostate radiotherapy in a contemporary cohort remains unclear [2].

CYTOREDUCTIVE RADICAL PROSTATECTOMY

Historical data from the Southwest Oncology Group (SWOG) 8894 trial randomising 1,286 men with metastatic disease to bilateral orchidectomy with placebo or flutamide demonstrated, that a subgroup of men who underwent previous radical prostatectomy had a significantly reduced risk of death (HR 0.77, [95% CI 0.53–0.80]) [45]. Building on this, numerous retrospective series and registry data (e.g. Surveillance, Epidemiology, and End Results, SEER) have reported improved OS and CSS in men who undergo cytoreductive radical prostatectomy with low-burden or predominantly osseous disease [46-51].

At present, prospective evidence is limited to a 61-patient case-control study conducted by Heidenreich and colleagues [52]. Performed in men with <4 metastases and no visceral or extensive lymph node metastases, with a serum PSA level <1.0 ng/ml after neoadjuvant ADT, cytoreductive radical prostatectomy (CRP) and extended pelvic lymph node dissection (ePLND) led to a 12.1 month improvement in PFS compared to the control arm ADT alone (38.6 months vs 26.5 months; $p = 0.0032$) [52]. There were no reported Grade 4 or 5 Clavien-Dindo classification complications within this study, confirming the surgical feasibility reported in previous retrospective studies [46, 47].

Multiple confirmatory randomised (NCT01751438 [BST]; NCT03655886 [LoMP II]) and single-arm studies (TRoMbone; NCT02716974; NCT03298087) are ongoing with, or without, MDT in this cohort [48, 53-56].

CYTOREDUCTIVE MINIMALLY INVASIVE ABLATIVE THERAPY (MIAT)

With regard to cytoreductive MIAT, a single retrospective study evaluating whole-gland cryotherapy in 23 men with a favourable response to 6-months ADT (PSA <1.0 ng/ml), </= cT3a disease, and limited bony metastasis, reported a 10-month survival advantage when compared to a matched cohort with ADT alone (35 months vs 25 months; HR 0.21, [95% CI 0.09–0.45]; $p = 0.0027$) [57].

For peer review only - http://bmjopen.bmj.com/site/about/guidelines.xhtml

Furthermore, the NCT02489357 pilot study interrogated the ‘cryo-immunological response’ with PD-1 blockade using the antibody (Pembrolizumab) in addition to cytoreductive cryotherapy [38]. In total 12 men with oligometastatic disease initiated 8-months of ADT and Pembrolizumab, with subsequent whole-gland cryotherapy [38]. Primary endpoint was PSA <0.6 ng/ml at 1 year, and this was met in 42% (*n* = 5). Median PFS was 14 months and median systemic therapy-free survival was 17.5 months [38]. There were no grade 3 adverse events, with Grade 1 (non-pad, occasional) urinary incontinence in 16.7% (*n* = 2) [38]. This profile is in keeping with the favourable early functional outcomes from cryotherapy in patients with non-metastatic disease [58]. With regard to safety there were no reported cases of rectal injury or fistulae in either study [38, 57]. In both studies men did not receive prior systemic therapy escalation and thus the ‘additive’ value of cytoreductive cryotherapy in such a cohort remains unclear [38, 57].

METASTASIS-DIRECTED THERAPY

In men with recurrent distant oligometastases, a number of early phase clinical trials (STOMP,ORIOLE, POPSTAR) have demonstrated promise with metastases-directed therapy (either SABR or metastatectomy), mainly with regards to improving ADT-free and early PFS [27, 59, 60]. It is unclear the impact of such interventions on OS [27, 59, 60].

In *de novo* oligometastatic disease, a single pilot study including 20 men underwent sequential systemic therapy (ADT), surgery (cytoreductive radical prostatectomy + PLND +/- RPLND), and consolidation SABR to visible bone metastasis [61]. A novel endpoint of “undetectable PSA (</= 0.05 ng/ml) following testosterone recovery” was used and achieved in 20% (*n* = 4). However, 95% (*n* = 19) achieved an undetectable PSA, irrespective of testosterone suppression, after all three treatments. The addition of SABR accounted for an undetectable PSA in 21% (4/19) of this sub-group of men, when treatments were analysed separately [61].

For peer review only - http://bmjopen.bmj.com/site/about/guidelines.xhtml

IP2-ATLANTA STUDY HYPOTHESIS

We hypothesise that men with metastatic disease who undergo treatment of the local tumour in the form of either radical therapy (prostatectomy or external beam radiotherapy), or minimally invasive ablative therapy, combined with metastases-directed therapy, will improve progression-free survival in comparison patients who receive standard of treatment alone.

PATIENT AND PUBLIC INVOLVEMENT

A patient-involvement focus group was held with six patients who had advanced or metastatic prostate cancer, to determine initial patient acceptability and gauge important opinions on the proposed amendment and study design. Four patients had previously received radiotherapy as either their primary or secondary treatment. Comments from the group discussion were recorded along with anonymous questionnaires, which the patients returned, by post after the meeting. Two patient and public involvement (PPI) representatives were present during the HRA REC assessment. They will continue to be involved throughout the duration of the trial with the Trial Management Group and other patients not involved in the direct management of the study will be on the independent Trials Steering Committee.

METHODS AND ANALYSIS

STUDY DESIGN

IP2-ATLANTA is an unblinded, randomised, multicentre, interventional three-arm study with an active comparator arm incorporating standard of care (SOC) (**Figure 1**). Study participants will be randomised to: Control arm (standard of care); Intervention arm 1 (MIAT +/- pelvic lymph node dissection [PLND]) or Intervention arm 2 (prostate external beam radiotherapy [EBRT] +/- PLNRT OR cytoreductive radical prostatectomy [CRP] +/- PLND). Systemic therapy in all arms includes ADT +/- Docetaxel, Abiraterone Acetate, Enzalutamide, Apalutamide, as appropriate. Men with low-burden disease in intervention arms are eligible for metastases-directed therapy in the form of SABR or surgery (**Figure 1**).

For peer review only - http://bmjopen.bmj.com/site/about/guidelines.xhtml

STUDY POPULATION

Men who are willing to undergo local therapy to the prostate and selective metastases-directed therapy for newly diagnosed metastatic prostate cancer in addition to standard care systemic treatment upfront.

ELIGIBILITY

INCLUSION CRITERIA

1. Diagnosed with prostate cancer within 6 months of screening visit
2. Metastatic disease (Any T, Any N, M1+) of any grade, stage or Prostate Specific Antigen (PSA) level.
3. Fit to undergo standard of care systemic treatment for metastatic disease and both minimally invasive therapy and prostate EBRT/cytoreductive radical prostatectomy.
4. Performance status 0-2.
5. Histologically-proven local tumour.

EXCLUSION CRITERIA

1. Patient did not undergo and/or is unable to undergo standard of care baseline imaging tests for confirmation of metastatic status (CT abdomen/pelvis AND chest X-ray (or CT chest) AND radioisotope bone scan (or whole body imaging such as MRI or PET imaging as alternative to all preceding scans mentioned here) AND prostate MRI.
2. Prior exposure to long-term androgen deprivation therapy or hormonal therapy for the treatment of prostate cancer unless started within 4 months of screening visit.
3. Prior chemotherapy or local or systemic therapy for treatment of prostate cancer (apart from ADT or hormonal therapy as outlined above).

IDENTIFICATION OF PATIENTS & CONSENT

All men diagnosed with prostate cancer who go to a multidisciplinary team meeting or a tumour board as well as any man meeting the eligibility criteria prior to tumour board discussion will be identified for screening. Members of the tumour board will identify patients suitable for IP2-ATLANTA. The treating clinicians will mention the study and then the local research nurses/fellows, clinical trial coordinators, clinical trial

For peer review only - http://bmjopen.bmj.com/site/about/guidelines.xhtml

practitioners or the treating clinicians will then approach the patient if they are interested. A Patient Information Sheet (PIS) will be given, or if agreed, emailed or posted out to the patient. Those patients already aware of the diagnosis can be approached by telephone to enquire as to their interest in the study so that a PIS can be then be sent out by email or post prior to a clinical visit. Patients will be given as much time as they need to read the PIS before consenting to participate (with a minimum of 24 hours).

For peer review only

RANDOMISATION

Stratified randomisation will take into account the following variables to create 16 strata in total:

- Intent to treat pelvic lymph nodes?: yes versus no
- Metastatic burden: low versus high (CHAARTED definition) [9].
- Intent to use systemic agent (i.e. Docetaxel, Abiraterone Acetate, Enzalutamide, Apalutamide)?: yes versus no
- Intent to use metastases directed therapy?: yes versus no

TRIAL TREATMENT

CONTROL ARM (STANDARD OF CARE)

SOC treatment as determined by the treating physician: ADT with or without Docetaxel or other systemic standard of care treatment, including but not limited to, Abiraterone Acetate or Enzalutamide. Radiotherapy to the prostate only in this arm is defined as for 'symptom control' in high volume (>/=4) metastases or to mirror current accepted local radiotherapy dose regimens, external beam prostate radiotherapy using a dose of 36Gy/6Fr OR 55Gy/20Fr OR 60Gy/20Fr over 27 days as defined in Local Radiotherapy Standard Operating Procedure (SOP), for men with low volume metastases (<4 metastases). MDT will not be permitted in the control arm. Palliative radiotherapy for symptom control or for prevention of fracture will be permitted as local clinical practice.

ADT, but not Docetaxel, may be initiated prior to recruitment. The decision as to which SOC systemic therapy regimen will be decided by the treating clinician and/or

For peer review only - http://bmjopen.bmj.com/site/about/guidelines.xhtml

clinical team, to be declared upfront prior to randomisation. If radiotherapy is planned for local disease in men randomised to the SOC arm with low volume metastases, then this will be declared prior to randomisation by the treating physician. The use of PLNRT will not be permitted in the control arm.

INTERVENTION ARMS

Whilst discussing the intervention arms we should also consider the impact that SOC treatment may have on down-staging the local tumour. As the SOC treatment would be administered prior to any local MIAT or CRP/EBRT in the intervention arms 1 and 2, an attempt at reclassifying the residual disease with a prostate MRI and biopsies would be pragmatic. This is to prevent patients from developing adverse events from unnecessary local treatment when they have no evidence of residual disease. Patients with positive post-SOC biopsies would then receive the local treatment as outlined below. Randomisation would occur at enrolment with planned intention to treat (ITT) and per-protocol analyses.

Biopsies at 6-9 months from initiation of SOC therapy are part of the protocol during the embedded feasibility pilot. As we currently do not know the significance of residual disease after SOC therapy, even if determined to be low-volume and low-grade, all patients with positive biopsies will be offered local treatment as per randomisation. The pilot stage would obtain a point estimate of the magnitude of this response and also patient acceptability of a post-systemic therapy prostate biopsy. Taken collectively this will assist in informing the study investigators of their ongoing utility in the main phase II component, where they are not presently mandated.

INTERVENTION ARM 1: MIAT

MIAT to the prostate with or without PLND in addition to SOC systemic treatment. The exact treatment protocol and modality used (cryotherapy or high-intensity focused ultrasound [HIFU]) will be set within the trial MIAT SOP. For those patients who are undergoing MIAT no local prostate radiotherapy will be given as part of the intervention. Radiotherapy can be administered for palliative reasons. Cases may be referred for multidisciplinary discussion to the ATLANTA MIAT Quality Assurance Board.

For peer review only - http://bmjopen.bmj.com/site/about/guidelines.xhtml

INTERVENTION ARM 2: RADICAL THERAPY

In addition to SOC systemic treatment, radical therapy involves either: i) cytoreductive radical prostatectomy, with or without PLND, or ii) prostate EBRT, with or without simultaneous PLNRT. The actual modality will be based on physician and patient preference as well as patient co-morbidities and performance status.

The surgical technique is at the discretion and expertise of the surgical team but will reflect current UK surgical practice, laid down in the cytoreductive radical prostatectomy SOP. Trial surgeons must meet minimum case volume and optimal complication outcomes prior to operating in this trial. Further, they must receive peer-approval from the IP2-ATLANTA Surgeons Quality Assurance Board. For patients who are undergoing prostatectomy no local prostate radiotherapy will be given as part of the intervention. Radiotherapy can be given subsequently for palliative reasons.

Two local prostate radiotherapy dose and fractionation options are available:

- **60Gy in 20 fractions**. Treating the prostate to 60Gy and the seminal vesicles to 47Gy using a simultaneous integrated boost administered over 27 days. If the pelvic lymph nodes are to be treated then this will be done simultaneously to a dose of 47Gy in 20 fractions (if treated).
- **74-78Gy in 37-39 fractions**. Treat the prostate to 74-78Gy and the seminal vesicles to 60Gy, using as simultaneous integrated boost. If the pelvic lymph nodes are to be treated then this will done simultaneously to a dose of 55Gy in 37-39 fractions (in accordance to same fractions employed for treating the prostate and seminal vesicles).

The principles of pelvic nodal treatment within the study will follow those of the PIVOTALboost study (nodal arm-B), with variation to allow both dose and fractionation regimes [62]. Quality assurance for radiotherapy will be completed by the UK national Radiotherapy Trials Quality Assurance (RTTQA) team.

METASTASES-DIRECTED THERAPY: INTERVENTION ARMS 1 & 2

In men with low-burden disease in both intervention arms 1 and 2, MDT, may be used but intent-to-use MDT is to be declared prior to randomisation. In the case of a metastatic recurrence after MDT, a re-treatment with MDT would be allowed if there

For peer review only - http://bmjopen.bmj.com/site/about/guidelines.xhtml

were new metastatic areas/locations. The imaging reporting of metastases as well as doses and protocol for MDT will be defined and determined by the Imaging Reporting SOP and Metastases-Directed Therapy SOP.

SABR should not be delivered whilst concurrent chemotherapy is being delivered. Concurrent hormonal therapy is acceptable. SABR delivered to metastases must be completed within 3 months of: prostate radiotherapy +/- PLNRT OR cytoreductive radical prostatectomy +/- PLND OR MIAT +/- PLND. Constraints on the dose and fractionations by anatomical site mirror all those defined in the SABR UK consortium guidelines v.6.1 guidelines (2019) or, if absent, in CORE Trial Radiotherapy, Planning & Delivery guidelines v.2.0 (2018) [63, 64]. Quality assurance for SABR will be completed by the RTTQA body.

STUDY ENDPOINTS & OUTCOME MEASURES

Primary and secondary endpoints for the study are presented in **Table 1**. Study outcome measures are presented in **Table 2**.

FOLLOW-UP

Follow-up will consist of 12-weekly serum PSA tests in the first year and 2-weekly thereafter, until mortality or 4 years after initial randomisation, whichever is first (**Table 3.**). PROMS will be collected every 6-months in the first year and annually thereafter, until mortality or 4 years after initial randomisation, whichever is first. Minimum follow-up for each patient will be 2 years. However, yearly follow-up will continue long term alongside linkage to national database.

PATIENT REPORTED OUTCOME MEASURES

The European Organisation for the Research and Treatment of Cancer (EORTC), Core Quality of Life Questionnaire-C30 (QLQ-C30), with prostate-specific, fatigue, elderly, general and bone metastases modules, International Prostatic Symptoms Score (IPSS), The Expanded Prostate Cancer Index Composite Bowel and Bladder (EPIC) and International Index of Erectile Function 5 (IIEF15) will be used. The EuroQol (EQ-5D-5L) will be used in the study as a generic measure of health-related

For peer review only

quality-of-life which can be linked to public preferences. These data will be used to calculate quality-adjusted life-years as part of a future health economic evaluation. Patients agreeing to return questionnaires on quality-of-life will continue to complete quality of life data for 4 years after enrolment.

STUDY VISITS

Follow up visits for the administration of SOC therapy and clinical review will occur in accordance with the local hospitals follow-up protocols. In the intervention arms, of the embedded feasibility pilot only, a prostate MRI, systematic and targeted transrectal or transperineal biopsy will be performed at 6-9 months following the initiation of the SOC therapy. In the main phase II component, these procedures can be carried out by local centres at the discretion of local clinicians and when they are, data should be collected on their outcomes.

For those randomised to MIAT or CRP/EBRT, a date for treatment(s) will be booked in accordance with the local hospital waiting lists. Removal of the urethral catheter after MIAT or CRP will occur after a minimum period of 7-days during a hospital visit or can be removed either at the GP surgery or at a local hospital to the patient.

Further clinical reviews will occur as SOC visits at 12, 26, 28, 32, 34 and 52 weeks in the 1st year and 24-weekly intervals thereafter until mortality or 2 years after enrolment, whichever is first.

Collection of partial postcodes

In order to get an area-based estimate of deprivation, the participants' partial postcodes will be converted into an Index of Multiple Deprivation (IMD) score. The IMD is the established index of deprivation for England and Wales and has been adopted widely in studies across local and national government. IMD is based on detailed ward-level index based on several separate domains. Similarly, Scottish Index of Multiple Deprivation (SIMD) will be collected for participants in Scotland. Additionally, partial postcodes will be collected only from the participants who provide optional consent. Data will be stored in the secure and password protected InForm Database.

For peer review only - http://bmjopen.bmj.com/site/about/guidelines.xhtml

Further Follow-up Imaging

Follow-up imaging to determine response from treatment on primary and metastatic disease will not be protocolled but we recommend imaging to take place when there is suspicion of progression, such as patients with a rising PSA (i.e. biochemical failure). The appropriate imaging will be chosen as per the local hospital resources and policies. We envisage that the majority will perform a combination of a prostate MRI, bone scintigraphy, PET-CT/MRI, whole body MRI or CT chest/abdomen/pelvis.

Long-term outcomes

Patients will be consented for their details to be linked to national registries for survival information such as NHS Information Centre/Office of National Statistics (ONS) in England/Wales and General Register Office in Scotland, Hospital Episode Statistics (HES). This ONS-HES linkage however is an optional consent.

STATISTICAL ANALYSES AND SAMPLE SIZE CALCULATION

Embedded Feasibility and Pilot

We are seeking to determine whether the randomisation of men with metastatic disease is feasible and whether men are compliant to the therapy following randomisation. We aim to approach 80 patients from up to 17 centres in the UK over a 6-month period to allow us to estimate a 33% recruitment rate with 95% confidence interval width of approximately ± 10 percentage points.

Main phase II component

The study will have 80% power to detect a treatment difference with a hazard ratio 0.7 in favour of any of the intervention arms compared to the control at a two-sided 5.0% significance level. This is based on the assumption that the accrual period will be uniform over 24 months, that the follow-up period will be 24 months, and that the median progression-free survival is 37 months. This calculation may be adjusted depending on the compliance rate assessed during the feasibility stage. The overall sample size will be 918 participants considering a 5% loss to follow-up (291 participants per group, 873 participants for three arms). This will allow the detection of an effect size of 9.2% increase in progression free survival at 24 months.

For peer review only - http://bmjopen.bmj.com/site/about/guidelines.xhtml

For peer review only

ADVERSE EVENT REPORTING

The Common Terminology Criteria for Adverse Events (CTCAEv5.0) domain will be used to report adverse events [65].

DATA COLLECTION

The principal means of data collection from participant visits will be Electronic Data Capture (EDC) using the web-based InForm database. All study data will be entered into electronic Case Report Forms (eCRFs) in a database provided by the Sponsor. All eCRFs will be completed using de-identified data.

DATA MONITORING & ARCHIVING

A combined independent data monitoring and trial steering committee will meet twice a year. All trial documentation, including that held at participating sites and the trial coordinating centre, will be archived for a minimum of 10 years following the end of the study.

TRIAL FUNDING, ORGANISATION AND ADMINISTRATION

IP2-ATLANTA trial was approved by the HRA Wales REC 5 (19/WA0005). IP2-ATLANTA is funded by the Wellcome Trust (204998/Z/16/Z). The study will be monitored periodically by trial monitors to assess the progress of the study, verify adherence to the protocol, ICH GCP E6 guidelines and other national/international requirements and to review the completeness, accuracy and consistency of the data

DISCUSSION

IP2-ATLANTA is a multicentre, phase II, randomised controlled trial. The study will provide Level I evidence on oncological outcomes from prostate minimally invasive ablative therapy or radical therapy, in combination with metastasis-directed therapy, against standard of care treatment alone, in men with newly-diagnosed hormone sensitive metastatic prostate cancer. If either intervention arm is proven to provide significant oncological benefit this will have wide-reaching implications on the current standard of care paradigm.

For peer review only

CONCLUSION

IP2-ATLANTA addresses an important research gap in the role of sequential systemic, local cytoreductive and metastasis-directed interventions in men with newly-diagnosed metastatic prostate cancer.

TRIAL STATUS

IP2-ATLANTA is open to recruitment in 10 centres in England and Wales and expected to complete its embedded feasibility pilot phase by late 2020 [66].

Acknowledgements: We would like to thank all the participants, study PI, trial clinicians, research nurses, Imperial Clinical Trial Unit staff and other site staff who have been responsible for setting up, recruiting participants and collecting the data for the IP2-ATLANTA trial. Further we are grateful for the ongoing support of the Trial Management Group and our trial patient representative. Finally, we would like to thank the trial oversight provided by ICTU and our trial funder the Wellcome Trust.

Contributors: Conception and design of ATLANTA trial: HUA, MJC, MW, TTS, TD, AF, AS, VK, ST, MG, NS, JS, ED, FF, NKN. All authors have read and approved the final manuscript.

Funding: The trial was funded by the Wellcome Trust (204998/Z/16/Z)

Patient consent for publication: Not required.

Conflicts of Interest

Martin J. Connor's research is support by University College London Hospitals (UCLH) Charity and the Wellcome Trust.

Kamalram Thippu Jayaprakash is currently supported by a research grant from the UK National Institute of Health Research (NIHR) Clinical Research Network Eastern. He has received educational/ and travel grants from Bayer UK, Janssen Oncology, Pfizer; Roche, Takeda.

Hashim U. Ahmed's research is supported by core funding from the United Kingdom's National Institute of Health Research (NIHR) Imperial Biomedical Research Centre.

For peer review only - http://bmjopen.bmj.com/site/about/guidelines.xhtml

Ahmed currently receives funding from the Wellcome Trust, Prostate Cancer UK, MRC (UK), Cancer Research UK, Sonacare Inc., Trod Medical, and Sophiris Biocorp for trials in prostate cancer. Ahmed was a paid medical consultant for Sophiris Biocorp, Sonacare Inc. and BTG in the past 3 years.

All other authors have no declaration

References

1 Smittenaar CR, Petersen KA, Stewart K, et al. Cancer incidence and mortality projections in the UK until 2035, *Br J Cancer* 2016;115:1147-55 doi:10.1038/bjc.2016.304 [doi] [published Online First: October 25].

2 Connor MJ, Shah TT, Horan G, et al. Cytoreductive treatment strategies for de novo metastatic prostate cancer, *Nature Reviews Clinical Oncology* 2019:1-15.

3 James ND, Spears MR, Clarke NW, et al. Survival with newly diagnosed metastatic prostate cancer in the “docetaxel era”: data from 917 patients in the control arm of the STAMPEDE trial (MRC PR08, CRUK/06/019), *Eur Urol* 2015;67:1028-38.

4 Miyake H, Matsushita Y, Watanabe H, et al. Prognostic significance of time to castration resistance in patients with metastatic castration-sensitive prostate cancer, *Anticancer Res* 2019;39:1391-6.

5 Chi KN, Agarwal N, Bjartell A, et al. Apalutamide for metastatic, castration-sensitive prostate cancer. *N Engl J Med* 2019;381:13-24.

6 Davis ID, Martin AJ, Stockler MR, et al. Enzalutamide with standard first-line therapy in metastatic prostate cancer. *N Engl J Med* 2019;381:121-31.

7 James ND, Sydes MR, Clarke NW, et al. Addition of docetaxel, zoledronic acid, or both to first-line long-term hormone therapy in prostate cancer (STAMPEDE): survival results from an adaptive, multiarm, multistage, platform randomised controlled trial, *Lancet* 2016;387:1163-77 doi:10.1016/S0140-6736(15)01037-5 [doi] [published Online First: March 19].

8 Fizazi K, Tran N, Fein L, et al. Abiraterone plus prednisone in metastatic, castration-sensitive prostate cancer, *N Engl J Med* 2017;377:352-60.

9 Sweeney CJ, Chen Y, Carducci M, et al. Chemohormonal therapy in metastatic hormone-sensitive prostate cancer, *N Engl J Med* 2015;373:737-46.

10 Weichselbaum RR, Hellman S. Oligometastases revisited, *Nature reviews Clinical oncology* 2011;8:378.

11 Connor MJ, Winkler M, Ahmed HU. Survival in Oligometastatic Prostate Cancer - A New Dawn or the Will Rogers Phenomenon? *JAMA oncology* 2020;6:185-6.

For peer review only - http://bmjopen.bmj.com/site/about/guidelines.xhtml

12 Sita-Lumsden A, Dart DA, Waxman J, et al. Circulating microRNAs as potential new biomarkers for prostate cancer, *Br J Cancer* 2013;108:1925.

13 Selth LA, Townley SL, Bert AG, et al. Circulating microRNAs predict biochemical recurrence in prostate cancer patients, *Br J Cancer* 2013;109:641.

14 Alix-Panabières C, Riethdorf S, Pantel K. Circulating tumor cells and bone marrow micrometastasis, *Clinical Cancer Research* 2008;14:5013-21.

15 Lorente D, Olmos D, Mateo J, et al. Decline in circulating tumor cell count and treatment outcome in advanced prostate cancer, *Eur Urol* 2016;70:985-92.

16 Lilleby W, Stensvold A, Mills IG, et al. Disseminated tumor cells and their prognostic significance in nonmetastatic prostate cancer patients, *International journal of cancer* 2013;133:149-55.

17 Morgan TM, Lange PH, Porter MP, et al. Disseminated tumor cells in prostate cancer patients after radical prostatectomy and without evidence of disease predicts biochemical recurrence, *Clinical cancer research* 2009;15:677-83.

18 Weckermann D, Polzer B, Ragg T, et al. Perioperative activation of disseminated tumor cells in bone marrow of patients with prostate cancer, *J Clin Oncol* 2009;27:1549-56.

19 Comen E, Norton L, Massague J. Clinical implications of cancer self-seeding, *Nature reviews Clinical oncology* 2011;8:369.

20 Kim M, Oskarsson T, Acharyya S, et al. Tumor self-seeding by circulating cancer cells, *Cell* 2009;139:1315-26.

21 Nowak DG, Cho H, Herzka T, et al. MYC drives Pten/Trp53-deficient proliferation and metastasis due to IL6 secretion and AKT suppression via PHLPP2, *Cancer discovery* 2015;5:636-51.

22 Shiozawa Y, Pedersen EA, Havens AM, et al. Human prostate cancer metastases target the hematopoietic stem cell niche to establish footholds in mouse bone marrow, *J Clin Invest* 2011;121:1298-312.

23 Sleeman JP. The metastatic niche and stromal progression, *Cancer Metastasis Rev* 2012;31:429-40.

24 Kaplan RN, Riba RD, Zacharoulis S, et al. VEGFR1-positive haematopoietic bone marrow progenitors initiate the pre-metastatic niche, *Nature* 2005;438:820-7.

25 Psaila B, Lyden D. The metastatic niche: adapting the foreign soil, *Nature Reviews Cancer* 2009;9:285-93.

26 Gundem G, Van Loo P, Kremeyer B, et al. The evolutionary history of lethal metastatic prostate cancer, *Nature* 2015;520:353.

For peer review only - http://bmjopen.bmj.com/site/about/guidelines.xhtml

27 Ryan Phillips, William Yue Shi, Matthew Deek, et al. Outcomes of Observation vs Stereotactic Ablative Radiation for Oligometastatic Prostate Cancer. The ORIOLE Phase 2 Randomized Clinical Trial. *JAMA Oncology* 2020 [published Online First: Mar 26,].

28 Palma DA, Olson RA, Harrow S, et al. Stereotactic ablative radiation therapy for the comprehensive treatment of oligometastatic tumors (SABR-COMET): results of a randomized trial, *International Journal of Radiation Oncology• Biology• Physics* 2018;102:S3-4.

29 Golden EB, Demaria S, Schiff PB, et al. An abscopal response to radiation and ipilimumab in a patient with metastatic non–small cell lung cancer, *Cancer immunology research* 2013;1:365-72.

30 Strigari L, Mancuso M, Ubertini V, et al. Abscopal effect of radiation therapy: Interplay between radiation dose and p53 status, *Int J Radiat Biol* 2014;90:248-55.

31 Postow MA, Callahan MK, Barker CA, et al. Immunologic correlates of the abscopal effect in a patient with melanoma, *N Engl J Med* 2012;366:925-31.

32 Brooks ED, Chang JY. Time to abandon single-site irradiation for inducing abscopal effects, *Nature Reviews Clinical Oncology* 2018:1.

33 Kubo M, Satoh T, Ishiyama H, et al. Enhanced activated T cell subsets in prostate cancer patients receiving iodine-125 low-dose-rate prostate brachytherapy, *Oncol Rep* 2018;39:417-24.

34 Sabel MS. Cryo-immunology: a review of the literature and proposed mechanisms for stimulatory versus suppressive immune responses, *Cryobiology* 2009;58:1-11.

35 Ablin RJ, Soanes WA, Gonder MJ. Elution of in vivo bound antiprostatic epithelial antibodies following multiple cryotherapy of carcinoma of prostate, *Urology* 1973;2:276-9.

36 Ablin RJ, Soanes WA, Gonder MJ. Prospects for cryo-immunotherapy in cases of metastasizing carcinoma of the prostate, *Cryobiology* 1971;8:271-9.

37 Benzon B, Glavaris SA, Simons BW, et al. Combining immune check-point blockade and cryoablation in an immunocompetent hormone sensitive murine model of prostate cancer, *Prostate cancer and prostatic diseases* 2018;21:126-36.

38 Ross AE, Hurley PJ, Tran PT, et al. A pilot trial of pembrolizumab plus prostatic cryotherapy for men with newly diagnosed oligometastatic hormone-sensitive prostate cancer, *Prostate cancer and prostatic diseases* 2020;23:184-93.

39 Connor MJ, Winkler M, Ahmed HU. Cytoreductive cryotherapy for newly diagnosed oligometastatic hormone-sensitive prostate cancer, *Prostate Cancer and Prostatic Diseases* 2020:1-2.

For peer review only - http://bmjopen.bmj.com/site/about/guidelines.xhtml

40 Tosoian JJ, Gorin MA, Ross AE, et al. Oligometastatic prostate cancer: definitions, clinical outcomes, and treatment considerations, *Nature Reviews Urology* 2017;14:15.

41 Khoo V. New concepts in prostate cancer management: the conundrum of managing oligometastatic disease in prostate cancer—through the looking glass darkly, *Clin Radiol* 2019.

42 Parker CC, James ND, Brawley CD, et al. Radiotherapy to the primary tumour for newly diagnosed, metastatic prostate cancer (STAMPEDE): a randomised controlled phase 3 trial, *Lancet* 2018;392:2353-66 doi:S0140-6736(18)32486-3 [pii] [published Online First: December 01].

43 Boevé LM, Hulshof MC, Vis AN, et al. Effect on survival of androgen deprivation therapy alone compared to androgen deprivation therapy combined with concurrent radiation therapy to the prostate in patients with primary bone metastatic prostate cancer in a prospective randomised clinical trial: data from the HORRAD trial, *Eur Urol* 2019;75:410-8.

44 Gregucci F, Fiorentino A. Re: Liselotte MS Boevé, Maarten CCM Hulshof, André N. Vis, et al. Effect on Survival of Androgen Deprivation Therapy Alone Compared to Androgen Deprivation Therapy Combined with Concurrent Radiation Therapy to the Prostate in Patients with Primary Bone Metastatic Prostate Cancer in a Prospective Randomized Clinical Trial: Data from the HORRAD Trial. Eur Urol 2019; 75: 410-8. *Eur Urol* 2019;75:e129.

45 Thompson IM, Tangen C, Basler J, et al. Impact of previous local treatment for prostate cancer on subsequent metastatic disease, *J Urol* 2002;168:1008-12.

46 Jang WS, Kim MS, Jeong WS, et al. Does robot-assisted radical prostatectomy benefit patients with prostate cancer and bone oligometastases? *BJU Int* 2018;121:225-31.

47 Sooriakumaran P, Karnes J, Stief C, et al. A multi-institutional analysis of perioperative outcomes in 106 men who underwent radical prostatectomy for distant metastatic prostate cancer at presentation, *Eur Urol* 2016;69:788-94.

48 Sooriakumaran P. Testing radical prostatectomy in men with prostate cancer and oligometastases to the bone: a randomized controlled feasibility trial, *BJU Int* 2017;120:E8-E20.

49 Heidenreich A, Fossati N, Pfister D, et al. Cytoreductive radical prostatectomy in men with prostate cancer and skeletal metastases, *European urology oncology* 2018;1:46-53.

50 Culp SH, Schellhammer PF, Williams MB. Might men diagnosed with metastatic prostate cancer benefit from definitive treatment of the primary tumor? A SEER-based study, *Eur Urol* 2014;65:1058-66 doi:10.1016/j.eururo.2013.11.012 [doi] [published Online First: June 01].

For peer review only - http://bmjopen.bmj.com/site/about/guidelines.xhtml

51 Rusthoven CG, Jones BL, Flaig TW, et al. Improved survival with prostate radiation in addition to androgen deprivation therapy for men with newly diagnosed metastatic prostate cancer, *Journal of Clinical Oncology* 2016;34:2835-42.

52 Heidenreich A, Pfister D, Porres D. Cytoreductive radical prostatectomy in patients with prostate cancer and low volume skeletal metastases: results of a feasibility and case-control study, *J Urol* 2015;193:832-8.

53 Pienta K. A Study of Definitive Therapy to Treat Prostate Cancer (oligo-mets) NCT02716974 . ;2019.

54 Nickols N, Rettig M. NCT03298087 Systemic and Tumor-Directed Therapy for Oligometastatic Prostate Cancer.

55 Chapin BF, Mcguire SE, Wang X, et al. No title, *A prospective, multicenter, randomized phase II trial of best systemic therapy (BST) or BST plus definitive treatment (Surgery or Radiation) of the primary tumor in metastatic prostate cancer.* 2015.

56 Nicolaas L. NCT03655886. Testing radical prostatectomy in men with prostate cancer and oligometastases to the bone: a randomized controlled feasibility trial (LoMP II). 2018.

57 Sheng M, Wan L, Liu C, et al. Cytoreductive cryosurgery in patients with bone metastatic prostate cancer: A retrospective analysis, *Kaohsiung J Med Sci* 2017;33:609-15.

58 Shah TT, Peters M, Eldred-Evans D, et al. Early-Medium-Term Outcomes of Primary Focal Cryotherapy to Treat Nonmetastatic Clinically Significant Prostate Cancer from a Prospective Multicentre Registry, *Eur Urol* 2019.

59 Ost P, Reynders D, Decaestecker K, et al. Surveillance or metastasis-directed therapy for oligometastatic prostate cancer recurrence: a prospective, randomized, multicenter phase II trial, *Journal of Clinical Oncology* 2017;36:446-53.

60 Siva S, Bressel M, Murphy DG, et al. Stereotactic abative body radiotherapy (SABR) for oligometastatic prostate cancer: a prospective clinical trial, *Eur Urol* 2018;74:455-62.

61 O'Shaughnessy MJ, McBride SM, Vargas HA, et al. A pilot study of a multimodal treatment paradigm to accelerate drug evaluations in early-stage metastatic prostate cancer, *Urology* 2017;102:164-72.

62 Syndikus I. ISRCTN80146950. A phase III randomised controlled trial of prostate and pelvis versus prostate alone radiotherapy with or without prostate boost (PIVOTALBoost). 2019 [published Online First: March,].

63 Khoo V. Conventional Care Versus Radioablation (Stereotactic Body Radiotherapy) for Extracranial Oligometastases (CORE) NCT02759783. ;2019.

For peer review only - http://bmjopen.bmj.com/site/about/guidelines.xhtml

64 The Faculty of Clinical Oncology of The Royal College of Radiologists. Stereotactic Ablative Body Radiation Therapy (SABR): A Resource. Version 6.1. 2019.

65 U.S. DEPARTMENT OF HEALTH AND HUMAN SERVICES. Common Terminology Criteria for Adverse Events (CTCAE) Version 5.0. 2017.

66 Connor MJ, Shah TT, Sukumar J, et al. Initial experience of the adjuvant treatments to the local tumor for metastatic prostate cancer: Assessment of novel treatment algorithms, a multicenter, phase II randomized controlled trial (IP2-ATLANTA). *Journal of Clinical Oncology* 2020.

For peer review only

Table 1. Study primary and secondary endpoints

**PRIMARY ENDPOINT: Embedded Feasibility Pilot**	a) Recruitment, randomisation and compliance to allocation<br>b) Adverse events<br>c) Proportion of patients with pathological complete response on post systemic therapy prostate biopsy at 6-9 months.
**PRIMARY ENDPOINT: Phase II**	a) Progression-free survival (PFS)<br>*Defined as a composite outcome of biochemical failure; local progression; lymph node progression or bone metastases progression (new sites); or progression or development of new distant metastases, defined as lymph nodes outside the pelvis, bone or organ involvement or skeletal-related events confirmed as progression as in the STAMPEDE randomised study (**Assessment of Progression**; **Supplementary Material**) [42].*
**SECONDARY ENDPOINT: Phase II**	a) Adverse events and side-effect profile<br>b) Predictive factors for PFS and OS in each arm.<br>c) Effect on PFS or OS from varying radiotherapy dosage and schedules<br>d) Effect on PFS and OS stratified by volume and site for local and metastatic disease<br>e) Effect on PFS and OS stratified by the use of metastases directed therapy.<br>f) Effect on PFS using an alternative definition of failure, defined as a PSA increase of >/=25% and >/=2ng/mL if PSA was >/=2ng/mL from baseline, or a PSA increase of >/=25% if PSA was <2ng/mL at random assignment.<br>g) Effect on PFS using an alternative definition of local progression of a soft tissue metastatic lesion: defined as an increase of >/=20% in the largest tumour dimension with a minimum absolute increase of 5mm. Local progression of bone metastases to be assessed using MD Anderson Cancer Center–criteria with a >/=25% increase in the size of a measurable lesion on CT or a >/=25% increase in the size of ill-defined lesions on CT considered to be progression (1, 2).<br>h) Costs and resource utilisation for future cost-effectiveness analyses<br>i) In those men undergoing repeat biopsies after 6-9 months of standard of care systemic therapy, the proportion of patients with negative biopsies.<br>j) In those men undergoing repeat prostate/pelvic MRI after 6-9 months of standard of care systemic therapy, the proportion of patients with a negative prostate MRI for local tumour.<br>k) In those men undergoing repeat imaging (local prostate/pelvic and / or other body areas) after 6-9 months of standard of care systemic

For peer review only

For peer review only - http://bmjopen.bmj.com/site/about/guidelines.xhtml

	therapy, the proportion of patients with reduction on imaging of metastatic tumour deposits.

For peer review only

For peer review only - http://bmjopen.bmj.com/site/about/guidelines.xhtml

Table 2. Study outcome measures

**PRIMARY OUTCOMES: Embedded Feasibility Pilot**	a) Compliance to randomised arm<br>b) Recruitment and randomisation rate<br>c) Safety (adverse events)<br>d) Proportion of patients with complete pathological response on post SOC systemic therapy prostate biopsies at 6-9 months.
**PRIMARY OUTCOMES: Phase II**	a) Progression-free survival (PFS)
**SECONDARY OUTCOMES: Phase II**	a) Urinary, sexual and rectal side-effects<br>b) Patient-reported outcomes using validated questionnaires<br>c) Progression on PSA and imaging and impact of clinical features on progression<br>d) Health-related quality-of-life<br>e) Data on costs and resource utilisation for future cost-effectiveness analysis

For peer review only

Table 3. Study visit schedule

	**Screening**	**Treatment**					**Post-treatment**	**Follow-up**
**Visit**	**1**	**2**	**3**	**4**	**5**	**6**	**7**	**8 onwards**
Week (+/-)	0	12 (+/- 4)	26 (+/-12)	28 (+/- 12)	32 (+/- 12)	34 (+/- 12)	52 (+/- 4)	Every 24 weeks (+/- 4) (year 2-4)
Informed consent	X							
Inclusion & exclusion criteria	X							
Demography	X							
Medical history	X							
Vital signs/physical examination/clinical or subject assessment	X		X				As deemed necessary based on medical history and AE review	
PSA Blood Test	X	X	X			X	X	X
PROMS Questionnaires	X		X				X	X<br>(at 24 months only)
Review/reporting of patient AEs/SAEs<br>(may be performed via a face to face or telephone or email consultation)		X	X	X	X	X	X	X
Blood and urine tests including those for biobanking	X		X			X	At time of failure or at 24, 36, 48 months	
Randomisation	X[1]							
Standard of Care Therapy	X	X	X	X	X	X	X	X
Imaging Tests (Combination of but not limited to CT, X-rays, PET, MRI, Bone Scan)	If not already performed (SOC)		Recommended but not protocolled				As deemed necessary	
Prostate MRI	If not already performed (SOC)		X<br>Mandatory in Pilot only. During Main Phase can be conducted at clinician discretion within standard of care process				As deemed necessary	
Biopsy			X<br>Mandatory in Pilot only. During Main Phase can be conducted at clinician discretion within standard of care process				As deemed necessary	
Testosterone blood Test	X<br>(if available)		X<br>(recommended but not mandated)				As deemed necessary	
Treatment in intervention arms					X			
Removal of catheter						X		

Legend: [1]Randomisation should be performed within 7 days from screening visit

For peer review only - http://bmjopen.bmj.com/site/about/guidelines.xhtml

For peer review only

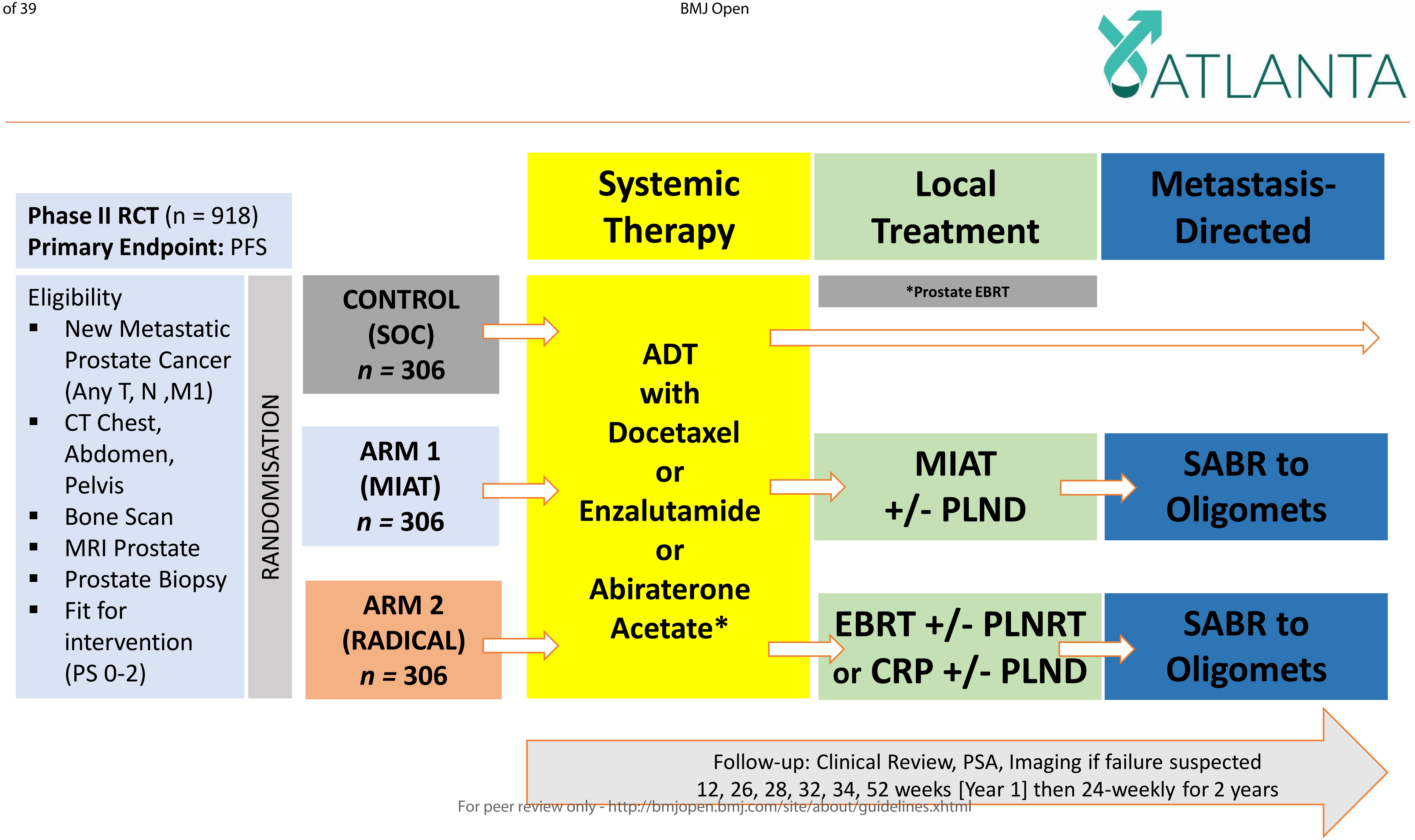

For peer review only - http://bmjopen.bmj.com/site/about/guidelines.xhtml

Supplementary Material
1.0 Assessment of progression

Baseline radiological examinations will be performed prior to enrolment. In the pilot only, prostate MRI and biopsy will be repeated at 26 (+/-12) weeks. In the main phase it is not protocol-mandated, that all patients have imaging scans repeated at the same time point (26 weeks +/- 12 weeks) and whenever clinically appropriate, such as in those with a low PSA value at enrolment or when there are concerns for progression i.e. biochemical failure, new bone pain, Skeletal Related Event (SRE). Clinicians can, at their own discretion, conduct a repeat MRI and biopsy of the prostate during the main phase.

The following outcomes should be reported:

- Biochemical failure
- Local progression
- Lymph node progression
- Bone metastases progression (new sites)
- Progression or development of new distant metastases, defined as lymph nodes outside the pelvis, bone or organ involvement
- Skeletal-related events confirmed as progression (see below).

Biochemical Failure

For the purposes of this trial, a unique threshold PSA value for biochemical failure is calculated, referred to as the PSA progression value. This value is derived for each patient based on their PSA nadir**,** defined as the lowest PSA value reported between randomisation and 6 months in the trial. The exact method for deriving the progression value for a patient depends on the value of their PSA nadir, and how this compares to their pre-treatment PSA value (i.e. the extent of the fall in PSA from the starting point).

The PSA progression value is calculated in one of three ways:

1. If the lowest recorded PSA value in the 26 weeks following randomisation is more than 4ng/ml and more than 50% of the pre-treatment PSA level then the patient fulfils the criteria for immediate treatment failure.
2. For patients whose PSA nadir in the 26 weeks following randomisation is less than or equal to 50% of the pre-treatment PSA level but remains above 4ng/ml, biochemical failure will be defined as a rise of 50% above the nadir level.
3. For patients whose PSA nadir is less than or equal to 4ng/ml, biochemical failure is defined as at least a 50% rise above the nadir value that is also above 4ng/ml.

Confirming biochemical failure: The timing and thus assessment of PSA needs to be considered because rises in PSA can occur due to non-cancer related causes such as after procedures, biopsies or urinary tract infection (UTI's). Confirmatory samples are needed in all cases of a rising PSA, prior to assigning an outcome of biochemical failure. After biochemical failure is confirmed for the first time it need not be reported again.

In the case that the raised PSA value reaches the progression value, a confirmatory PSA test should be performed after at least 1 week or 4 weeks after the completion of treatment in cases of UTI's, procedures or biopsies. Biochemical failure is confirmed

For peer review only

if the second value is around the same level or higher. The date of PSA progression should be provided as the date of the **first** raised PSA that fulfilled the definition for progression.

A confirmatory PSA is not required if there are other signs of progression e.g. progression of cancer related symptoms (clinical progression) or new radiological progression.

Second line treatment commenced specifically for biochemical failure should not start until the trial definition for biochemical failure has been met. However, if second line treatment does start before the trial definition is met then report the closest PSA value prior to the treatment start date as the progression value. This is not required if second line treatment is being started for other signs of progression e.g. clinical or radiological.

Testosterone levels: are required when reporting biochemical progression whilst receiving hormone treatment to confirm the diagnosis of castrate resistant prostate cancer.

Local, Lymph Node and Metastatic Failure
For each of local, lymph node and distant metastases progression, **both** the following should be reported:

- Date of first clinical/symptomatic progression
- Date of first objective/radiological progression.

Skeletal-related Events
Skeletal-related events (SREs) are defined as:

- Pathological fracture
- Spinal cord compression
- Requirement for RT to bone (e.g. for pain or impending fracture)
- Requirement for surgery (e.g. for prevention or management of fracture).

All SREs should be investigated further to establish whether or not the patient has progressed and only logged as progression if confirmed clinically or on imaging to be due to metastatic prostate cancer.

For peer review only

2.0 Study Management

All trial documentation, including that held at participating sites and the trial coordinating centre, will be archived for a minimum of 10 years following the end of the study. Subject files and other source data (including copies of protocols, CRFs, original reports of test results, correspondence, records of informed consent, and other documents pertaining to the conduct of the study) must be retained. Documents should be stored in such a way that they can be accessed/data retrieved later. Consideration should be given to security and environmental risks.

No study document will be destroyed without prior written agreement between the Sponsor and the investigator. Should the investigator wish to assign the study records to another party or move them to another location, written agreement must be obtained from the Sponsor.

Storage and handling of confidential trial data and documents will be in accordance with the Data Protection Act 2018 (UK).

Study Management Structure

A Trial Steering Committee (TSC) will be convened including as a minimum an Independent Chair, Independent Clinician, the Chief Investigator and Study Manager. The role of the TSC is to provide overall supervision of trial conduct and progress. Details of membership, responsibilities and frequency of meetings will be defined in a separate Charter.

A Trial Management Group (TMG) will be convened including the Chief Investigator, co-investigators and key collaborators, Study Statistician and Study Manager. The TMG will be responsible for day-to-day conduct of the trial and operational issues. Details of membership, responsibilities and frequency of meetings will be defined in separate Terms of Reference. When necessary, decisions will be referred to the TSC. Meetings will be scheduled in a risk-adapted manner to allow for the review of events during the trial.

A combined data monitoring and trial steering committee will meet twice a year basis. The composition of this committee will include but not be limited to the Chief Investigator, Trial Statistician, Trial Coordinator, Trials Unit representative, Research nurse and Patient representative

In case of early discontinuation of the study, the Follow-up Visit assessments should be performed for each subject, as far as possible.

The following reasons may result in early discontinuation:

- Early evidence that a treatment arm is harmful. If only one treatment arm is deemed to be harmful then the remaining arms of the study may continue as planned,

OR

- It is not feasible to reach the planned outcomes (A hazard ration of 0.7 to 1 will make the intervention not worth progressing with given the severe adverse effects associated with it)

For peer review only

The statistical criteria for termination of the study will be detailed in the statistical analysis plan (SAP).

A study-specific risk assessment will be performed prior to the start of the study to assign a risk category of ‘low’, ‘medium’ or ‘high’ to the trial. Risk assessment will be carried out by the ICTU QA Manager in collaboration with the Study Manager and the result will be used to guide the monitoring plan. The risk assessment will consider all aspects of the study and will be updated as required during the course of the study. The study will be monitored periodically by trial monitors to assess the progress of the study, verify adherence to the protocol, ICH GCP E6 guidelines and other national/international requirements and to review the completeness, accuracy and consistency of the data.

Monitoring procedures and requirements will be documented in a Monitoring Plan, developed in accordance with the risk assessment.

Quality Control will be performed according to ICTU/ internal procedures. The study may be audited by a Quality Assurance representative of the Sponsor and/or ICTU. All necessary data and documents will be made available for inspection.

The study may be subject to inspection and audit by regulatory bodies to ensure adherence to GCP and the NHS Research Governance Framework for Health and Social Care (2nd Edition).

Dissemination of findings

Information concerning the study, patent applications, processes, scientific data or other pertinent information is confidential and remains the property of the Sponsor. The investigator may use this information for the purposes of the study only.

It is understood by the investigator that the Sponsor will use information developed in this clinical study in connection with the development of the ablative or radiotherapy or surgical techniques and, therefore, may disclose it as required to other clinical investigators and to Regulatory Authorities. In order to allow the use of the information derived from this clinical study, the investigator understands that he/she has an obligation to provide complete test results and all data developed during this study to the Sponsor.

Verbal or written discussion of results prior to study completion and full reporting should only be undertaken with written consent from the Sponsor.

Therefore all information obtained as a result of the study will be regarded as CONFIDENTIAL, at least until appropriate analysis and review by the investigator(s) are completed.

Permission from the Executive/Writing Committee is necessary prior to disclosing any information relative to this study outside of the Trial Steering Committee. Any request by site investigators or other collaborators to access the study dataset must be formally reviewed by the TMG.

For peer review only

The results may be published or presented by the investigator(s), but the Funder will be given the opportunity to review and comment on any such results for up to 1 month before any presentations or publications are produced.

A Clinical Study Report summarising the study results will be prepared and submitted to the REC within a year of the end of study.

For peer review only

3.0 Informed Consent Form

ATLANTA

Additional Treatments to the Local tumour for metastatic prostate cancer: Assessment of Novel Treatment Algorithms

INFORMED CONSENT FORM

Chief Investigator: Professor Hashim U. Ahmed

Principal Investigator: <<Insert Principal Investigator>>

Please initial each box below. Do not tick

1	I confirm that I have read and understand the patient information sheet dated _ _ / _ _ / _ _ _ _ (Version ___) for the **ATLANTA** Study. I have had the opportunity to consider the information, ask questions and have had these answered satisfactorily.	
2	I understand that the type of treatment I receive will be allocated using a randomisation process, and neither myself nor the staff involved in the study can influence this allocation.	
3	I understand that if at any point my medical condition changes, it may be necessary to withdraw from the trial and have treatment options reviewed. This will be discussed with me by clinicians and with my agreement.	
4	I understand that I may be asked questions relating to personal aspects such as about diet and lifestyle from my local research team.	
5	I understand that my participation is voluntary and that I am free to withdraw at any time, without giving reason and without my medical care or legal rights being affected.	
6	I understand that relevant sections of my medical notes and data collected during the study, may be looked at by individuals from the Sponsor of the trial (Imperial College London) and responsible persons authorised by the Sponsor, from regulatory authorities or from the NHS Trust, where it is relevant to my taking part in this research. I give permission for these individuals to have access to my records.	
7	I understand that the information collected about me will be used to support other research in future, and may be shared anonymously with other researchers.	
8	I give permission for all standard of care data and samples, including those taken prior to study recruitment, such as surgery specimen and biopsies including tissue, bloods, urine and imaging tests to be used in this study even if I withdraw at any points from the study	

For peer review only

9	I give permission for the researchers to contact me regarding this trial during this trial period.	
10	I agree for my GP and other doctors to be informed of my participation in this study and of any clinical relevant study results	
11	I agree to take part in the above study.	

All the boxes above must be initialled for consent to be valid

Please initial each box below. Do not tick

OPTIONAL		
12	I give permission for additional blood and urine samples to be taken and be made available for future research where the samples would be stored appropriately and the research approved separately ***(If you do not wish to give this permission, do not initial – you can still participate in the study).***	
13	I give permission for all standard of care samples and data such as surgery specimen, biopsies including tissue as well as imaging scans to be made available for future research where the samples and scans would be stored appropriately and the research approved separately ***(If you do not wish to give this permission, do not initial – you can still participate in the study).***	
14	I give permission for any blood, urine and tissue samples, which will look for changes in my genetic material (DNA) as described in the information sheet, to be used in this study. ***(If you do not wish to give this permission, do not initial – you can still participate in the study).***	
15	I give permission for any blood, urine and tissue samples, which will look for changes in my genetic material (DNA) as described in the information sheet, to be used for further ethically approved research in the field of prostate cancer research. ***(If you do not wish to give this permission, do not initial – you can still participate in the study).***	
16	I give permission for my samples and data from any scans to be sent and utilised in research both in the UK and worldwide. All material will be anonymous and I will not be identifiable. I understand that I will not be asked again for permission to run these additional research tests and I may also not be informed of the results ***(If you do not wish to give this permission, do not initial – you can still participate in the study).***	
17	I give permission for the researchers to contact me in the future regarding the possibility of further studies, but I understand that I am under no obligation to take part in these ***(If you do not wish to give this permission, do not initial – you can still participate in the study).***	
18	I give permission for all samples taken to be biobanked and transferred to the Imperial College Healthcare Tissue Bank (ICHTB) or other UK-based biobank for a period of up to 10 years (or per local policy) and will be used for histological, genomic and epigenetic analysis and for ethically approved future	

For peer review only

	studies by our team or other scientists interested in prostate cancer research. Samples will not have any personal information written on them. Researchers will not be able to identify you from your samples. ***(If you do not wish to give this permission, do not initial – you can still participate in the study).***	
19	I give permission for my name and NHS number to be used to obtain information about my health status from records held by the NHS and maintained by the NHS Information Centre and the NHS Central Register or any applicable NHS information system (including linkage to routine hospital admission data). I give this consent solely so that researchers may follow up on my health status for 10 years after my participation in the study. ***(If you do not wish to give this permission, do not initial – you can still participate in the study).***	
20	I give permission for my partial postcode to be recorded and stored. This will be used for research purposes only and will remain confidential. ***(If you do not wish to give this permission, do not initial – you can still participate in the study****).*	

Name of Participant Signature Date

________________________ ________________________ ________________

Name of Person taking consent Signature Date

________________________ ________________________ ________________

Please give one copy of the consent form to the patient, file one copy in the patient's medical records, and retain the original in the Investigator Site File

For peer review only

BMJ Open

Additional Treatments to the Local tumour for metastatic prostate cancer - Assessment of Novel Treatment Algorithms (IP2-ATLANTA): Protocol for a multicentre, phase II randomised controlled trial

Journal:	*BMJ Open*
Manuscript ID	bmjopen-2020-042953.R1
Article Type:	Protocol
Date Submitted by the Author:	08-Jan-2021
Complete List of Authors:	Connor, Martin; Imperial College London, Department of Surgery and Cancer; Imperial College Healthcare NHS Trust, Imperial Urology<br>Shah, Taimur T; Imperial College London, Department of Surgery and Cancer<br>Smigielska, Katarzyna; Imperial College London, Department of Surgery and Cancer; Imperial College London, Imperial College Clinical Trials Unit<br>Day, Emily; Imperial College London, Imperial College Clinical Trials Unit<br>Sukumar, Johanna; Imperial College London, Department of Surgery and Cancer; Imperial College London, Imperial College Clinical Trials Unit<br>Fiorentino, Francesca; Imperial College London, Imperial College Clinical Trials Unit<br>Sarwar, Naveed; Imperial College Healthcare NHS Trust, Department of Oncology<br>Gonzalez, Michael; Imperial College Healthcare NHS Trust, Department of Oncology<br>Falconer, Alison; Imperial College Healthcare NHS Trust, Department of Oncology<br>Klimowska-Nassar, Natalia; Imperial College London, Department of Surgery and Cancer; Imperial College London, Imperial College Clinical Trials Unit<br>Evans , Martin; Imperial College London, Department of Surgery and Cancer<br>Naismith, Olivia; Royal Marsden NHS Foundation Trust, Radiotherapy Trials Quality Assurance (RTTQA)<br>Thippu Jayaprakash, Kamalram; Addenbrooke's Hospital, Department of Oncology<br>Price, Derek; Imperial College London, Department of Surgery and Cancer<br>Gayadeen, Shiva; Imperial College Healthcare NHS Trust, Department of Oncology<br>Basak, Dolan; Imperial College Healthcare NHS Trust, Department of Oncology<br>Horan, Gail; Addenbrooke's Hospital, Department of Oncology<br>McGrath, John; Royal Devon and Exeter NHS Foundation Trust, Urology<br>Sheehan, Denise; Royal Devon and Exeter NHS Foundation Trust, Department of Oncology<br>Kumar, Manal; Arrowe Park Hospital, Department of Urology<br>Ibrahim , Azman; Clatterbridge Cancer Centre NHS Foundation Trust,

	Department of Clinical Oncology<br>Brock, Cathryn; Chelsea and Westminster Hospital NHS Foundation Trust, Department of Oncology<br>Pearson, Rachel; Newcastle Upon Tyne Hospitals NHS Foundation Trust, Department of Oncology<br>Anyamene , Nicola; London North West University Healthcare NHS Trust, Department of Oncology<br>Heath , Catherine; University Hospital Southampton NHS Foundation Trust, Department of Radiotherapy<br>Shergill, Iqbal; Wrexham Maelor Hospital, Department of Urology<br>Rai, Bhavan; Newcastle Upon Tyne Hospitals NHS Foundation Trust, Department of Urology<br>Hellawell, Giles; London North West University Healthcare NHS Trust, Department of Urology<br>Mccracken, Stuart; Sunderland Royal Hospital, Department of Urology<br>Khoubehi, Bijan; Chelsea and Westminster Healthcare NHS Trust, Department of Urology<br>Mangar, Stephen; Imperial College Healthcare NHS Trust, Department of Oncology<br>Khoo, Vincent; Royal Marsden NHS Foundation Trust, Department of Oncology<br>Dudderidge, Tim; University Hospital Southampton NHS Foundation Trust, Department of Urology<br>Staffurth, John; Velindre Cancer Centre, Research; Cardiff University School of Medicine, Division of Cancer and Genetics<br>Winkler, Mathias; Imperial College London, Department of Surgery and Cancer; Imperial College Healthcare NHS Trust, Imperial Urology<br>Ahmed, Hashim; Imperial College London Division of Surgery, Imperial College London
**Primary Subject Heading**:	Urology
Secondary Subject Heading:	Oncology, Surgery, Research methods
Keywords:	Prostate disease < UROLOGY, Radiation oncology < RADIOTHERAPY, RADIOTHERAPY, UROLOGY, Urological tumours < UROLOGY, Genitourinary imaging < RADIOLOGY & IMAGING

I, the Submitting Author has the right to grant and does grant on behalf of all authors of the Work (as defined in the below author licence), an exclusive licence and/or a non-exclusive licence for contributions from authors who are: i) UK Crown employees; ii) where BMJ has agreed a CC-BY licence shall apply, and/or iii) in accordance with the terms applicable for US Federal Government officers or employees acting as part of their official duties; on a worldwide, perpetual, irrevocable, royalty-free basis to BMJ Publishing Group Ltd ("BMJ") its licensees and where the relevant Journal is co-owned by BMJ to the co-owners of the Journal, to publish the Work in this journal and any other BMJ products and to exploit all rights, as set out in our licence.

The Submitting Author accepts and understands that any supply made under these terms is made by BMJ to the Submitting Author unless you are acting as an employee on behalf of your employer or a postgraduate student of an affiliated institution which is paying any applicable article publishing charge ("APC") for Open Access articles. Where the Submitting Author wishes to make the Work available on an Open Access basis (and intends to pay the relevant APC), the terms of reuse of such Open Access shall be governed by a Creative Commons licence – details of these licences and which Creative Commons licence will apply to this Work are set out in our licence referred to above.

Other than as permitted in any relevant BMJ Author's Self Archiving Policies, I confirm this Work has not been accepted for publication elsewhere, is not being considered for publication elsewhere and does not duplicate material already published. I confirm all authors consent to publication of this Work and authorise the granting of this licence.

For peer review only

Additional Treatments to the Local tumour for metastatic prostate cancer - Assessment of Novel Treatment Algorithms (IP2-ATLANTA): Protocol for a multicentre, phase II randomised controlled trial

Martin J. Connor [1,2,3], Taimur T. Shah [1], Katarzyna Smigielska [1,4], Emily Day [1,4], Johanna Sukumar [1, 4], Francesca Fiorentino [1,4], Naveed Sarwar [5], Michael Gonzalez [5], Alison Falconer [5], Natalia Klimowska-Nassar [1, 4], Martin Evans [1, 4], Olivia Frances Naismith [6], Kamalram Thippu Jayaprakash [7], Derek Price [1], Shiva Gayadeen [5], Dolan Basak [5], Gail Horan [7], John McGrath [8], Denise Sheehan [8], Manal Kumar [9], Azman Ibrahim [10], Cathryn Brock [11], Rachel A. Pearson [12], Nicola Anyamene [13], Catherine Heath [14], Iqbal Shergill [15], Bhavan Rai [12], Giles Hellawell [13], Stuart McCracken [16], Bijan Khoubehi [11], Stephen Mangar [5], Vincent Khoo [17], Tim Dudderidge [14], John Nicholas Staffurth [18], Mathias Winkler [1,2,3], Hashim U. Ahmed [1,2]

1. Division of Surgery, Imperial Prostate, Department of Surgery and Cancer, Faculty of Medicine, Imperial College London, London, UB1 3HW
2. Imperial Urology, Imperial College Healthcare NHS Trust, London, W6 8RF
3. Department of Urology, West Middlesex University Hospital (WMUH), Chelsea & Westminster NHS Foundation Trust, Isleworth, Middlesex, TW7 6AF
4. Imperial Clinical Trials Unit (ICTU), School of Public Health, Faculty of Medicine, Imperial College London, London, W12 7RH
5. Department of Oncology, Imperial College Healthcare NHS Foundation Trust, Charing Cross Hospital, London, W6 8RF
6. Radiotherapy Trials Quality Assurance (RTTQA), Department of Physics, The Royal Marsden NHS Foundation Trust, London, SW3 6JJf
7. Department of Oncology, Addenbrooke's Hospital and The Queen Elizabeth Hospital Kings Lynn NHS Foundation, Norfolk, PE30 4ET
8. Department of Urology & Oncology, Royal Devon & Exeter Hospital, Exeter EX2 5DW

For peer review only - http://bmjopen.bmj.com/site/about/guidelines.xhtml

9. Department of Urology, Arrowe Park Hospital, Wirral University NHS Foundation Trust, Merseyside, CH49 5PE
10. Department of Oncology, Clatterbridge Cancer Centre NHS Foundation Trust, Wirral, CH63 4JY
11. Department of Urology & Oncology, Chelsea and Westminster Hospital, Chelsea & Westminster NHS Foundation Trust, SW10 9NH.
12. Department of Urology & Oncology, Northern Centre for Cancer Care, Newcastle upon Tyne Hospitals NHS Foundation Trust, NE7 7DN
13. Department of Urology & Oncology, Northwick Park Hospital, London Northwest University Healthcare NHS Trust, Harrow, HA1 3UJ
14. Department of Urology & Oncology, University Hospital Southampton NHS Foundation Trust, Southampton, SO16 6YD
15. Department of Urology, Wrexham Maelor Hospital, Wales, LL13 7TD
16. Department of Urology, Sunderland Royal Hospital, Sunderland, SR4 7TP
17. Department of Clinical Oncology, The Royal Marsden Hospital & Institute of Cancer Research, London, SW3 6JJ,
18. School of Medicine, Cardiff University, Cardiff, Wales, CF10 3AT

Corresponding Author:

Mr. Martin J. Connor

Division of Surgery

Department of Surgery & Cancer

Imperial College London

Room 5L.28

Charing Cross Lab Block

martin.connor2@nhs.net

Keywords: *prostate cancer, metastatic, radiotherapy, radical prostatectomy, cytoreductive, SABR, stereotactic radiotherapy, high intensity focussed ultrasound, cryotherapy, oligo-metastatic disease, metastasis directed therapy.*

For peer review only

Abstract

Introduction: Survival in men diagnosed with *de novo* synchronous metastatic prostate cancer has increased following the use of upfront systemic treatment, using chemotherapy and other novel androgen receptor targeted agents, in addition to standard androgen deprivation therapy (ADT). Local cytoreductive and metastasis-directed interventions are hypothesised to confer additional survival benefit. In this setting, IP2-ATLANTA will explore progression-free survival (PFS) outcomes with the addition of sequential multi-modal local and metastasis-directed treatments compared to standard care alone.

Methods: A phase II, prospective, multi-centre, three-arm randomised controlled trial incorporating an embedded feasibility pilot. All men with new histologically diagnosed, hormone-sensitive, metastatic prostate cancer, within four months of commencing ADT and of performance status 0 to 2 are eligible. Patients will be randomised to: Control (Standard of Care [SOC]) OR Intervention 1 (minimally invasive ablative therapy to prostate +/- pelvic lymph node dissection [PLND]) OR Intervention 2 (cytoreductive radical prostatectomy +/- PLND OR prostate radiotherapy +/- pelvic lymph node radiotherapy [PLNRT]). Metastatic burden will be pre-specified using the CHAARTED definition. Men with low burden disease in intervention arms are eligible for metastasis-directed therapy, in the form of stereotactic ablative body radiotherapy (SABR) or surgery. Standard systemic therapy will be administered in all arms With ADT +/- upfront systemic chemotherapy or androgen receptor agents. Patients will be followed-up for a minimum of two-years. Primary outcome: PFS. Secondary outcomes include: predictive factors for PFS and overall survival; urinary, sexual and rectal side-effects. Embedded feasibility sample size is 80, with 918 patients required in the main phase II component. Study recruitment commenced in April 2019, with planned follow-up completed by April 2024.

Ethics and dissemination: Approved by the Health Research Authority (HRA) Research Ethics Committee Wales-5 (19/WA0005). Study results will be submitted for publication in peer-reviewed journals.

Registration details: NCT03763253; ISCRTN58401737

For peer review only - http://bmjopen.bmj.com/site/about/guidelines.xhtml

STRENGTHS AND LIMITATIONS OF THIS STUDY

1. IP2-ATLANTA addresses an important research gap in the role of local and metastasis-directed therapy in men with newly-diagnosed metastatic prostate cancer.
2. This is the first phase II trial to include cytoreductive minimally invasive ablative therapy alongside cytoreductive radical prostatectomy and prostate radiotherapy.
3. The IP2-ATLANTA study builds on the clinical benefits derived from metastasis-directed therapy (stereotactic ablative body radiotherapy and/or surgery) in a previously untreated cohort of men with advanced disease.
4. Due to invasive interventions, blinding is not possible in the IP2-ATLANTA study.

For peer review only

For peer review only - http://bmjopen.bmj.com/site/about/guidelines.xhtml

INTRODUCTION

Overall, 47,000 men are diagnosed with prostate cancer each year in the United Kingdom (UK) [1]. Approximately, 4,500 of these men this will be diagnosed with *de novo* synchronous metastatic disease at presentation [1]. As with the United States, where just under 8% present with metastatic disease and where the annual burden is predicted to reach approximately 15,000 cases by 2025, so the prediction for the same magnitude is likely for the UK [2].

Traditionally, such men were managed with androgen deprivation therapy (ADT) alone, via medical or surgical castration [2]. Unfortunately the median time to the emergence of a castrate resistant state is in the order of 11 to 18 months, limiting overall survival (OS) to 3.5 years [3, 4]. Promisingly, the reported OS in this group has now risen to a median 4.8 years with the addition of upfront systemic agents, such as Docetaxel, Enzalutamide, Abiraterone Acetate or Apalutamide [5-9].

Moving beyond early systemic therapy escalation there has been an increased focus on the role of local cytoreductive and metastasis-directed interventions (primarily stereotactic ablative body radiotherapy [SABR]) to gain additional oncological benefit [2]. This is in part based upon the emergence of the 'oligo-metastatic state', which may exhibit different biological characteristics to poly-metastatic prostate cancer [10].

Such men present with a clinically-defined favourable metastatic burden, and are hypothesised to occupy an intermediate state between 'locally advanced' and 'poly-metastatic' disease [10, 11]. It is postulated, but not proven, that men exhibiting such disease patterns gain most benefit in progression-free survival (PFS) and OS resulting from localised cancer control achieved via cytoreductive interventions [2, 10].

IP2-ATLANTA STUDY HYPOTHESIS

We hypothesise that men with metastatic disease who undergo treatment of the local tumour in the form of either radical therapy (cytoreductive radical prostatectomy (CRP) or external-beam radiotherapy (EBRT)), or minimally invasive ablative therapy (MIAT), combined with metastases-directed therapy (MDT), will improve progression-free survival in comparison patients who receive standard of treatment alone.

For peer review only - http://bmjopen.bmj.com/site/about/guidelines.xhtml

PATHOBIOLOGICAL BASIS FOR LOCAL CYTOREDUCTIVE & METASTASIS DIRECTED THERAPY

The pathobiological basis underpinning local cytoreductive and metastasis-directed therapy in prostate cancer is not fully delineated[2]. Local prostate cytoreduction is thought to primarily impact on tumour-derived factors such as cytokines, chemokines and microRNAs [12, 13]. In particular, prostate tumour cell shedding and dissemination has been shown to occur earlier, with the detection of circulating tumour cells (CTCs) in blood and disseminated tumour cells (DTCs) in bone marrow of patients staged as non-metastatic on conventional imaging (i.e. bone scintigraphy) [14-18].

This has led to a comparison to the "self-seeding" hypothesis, as described in other solid organ malignancies involving the breast and colon [19, 20]. It posits that the return of CTCs or DTCs from distant secondary sites alters the primary tumour microenvironment (TME), via release of matrix metalloproteinases (e.g. matrix metalloproteinase-1) and cytokines (e.g. CXC-motif chemokine 1) [19, 20]. Such circulation may lead not only to "self-seeding" but also the remodelling of a "pre-metastatic niche" at new distant sites [19, 21, 22]. Bone marrow-derived haematopoietic cells localise to support pre-metastatic niche's, promoting the local environment for colonisation [14, 16] [22].

Furthermore, investigators utilising multifocal sequencing approaches have revealed the present of primary-tumour-to-metastasis, but also surprisingly, metastasis-to-metastasis transfer of clonal tumour cells [23]. This subsequently lead to the exploration of metastasis-directed therapy [24, 25]. Such interventions are hypothesised to have an effect on distant tumours via the release of tumour antigens, damage-associated molecular patterns (DAMPs), and local activation of immune cells (including cytotoxic T-cells) [26-29]. This has been coined the 'abscopal effect' and is associated with the generation of a systemic anti-tumour immune response. Evidence for such a response in prostate cancer remains sparse [24, 30].

Immune-mediate responses may not be limited to cytotoxic radiotherapy, with minimally invasive ablative therapy (MIAT), such as the 'cryo-immunological response' [31]. Similar to the cytotoxic abscopal response, the clinical translation of such

For peer review only - http://bmjopen.bmj.com/site/about/guidelines.xhtml

observed responses is unclear [2]. Early local prostate cryotherapy case series reported spontaneous distant regression of metastasis, although this has not been replicated in the contemporary literature [31, 32]. Furthermore, clinical augmentation of prostate cryotherapy by immune-checkpoint inhibitors (e.g. anti-programmed cell death-1 antibody, PD-1) also demonstrated pre-clinical promise, but proved disappointing when translated into early phase clinical studies [33, 34] [35].

When taken collectively, removal of the primary tumour and possibly its metastatic sites, may lead to a disruption in these immune-mediated pathological relationships and result in regression of metastases with a prolonged cancer-specific survival (CSS).

CATEGORISING METASTATIC BURDEN

A key research barrier at present is that there is no universally accepted definition for oligometastatic disease, which varies depending on the anatomical site (nodal, burden, visceral), absolute number (1 to 7), temporal pattern (outside vertebral bodies or pelvis) and diagnostic imaging utilised (conventional or molecular) [11]. Consequently there is also no accepted definition for 'high' versus 'low' volume disease [10, 36, 37]. At present oncology trials exploring systemic therapy have frequently adopted the use of the CHAARTED definition of metastatic disease burden. High burden disease is defined as four or more bone metastases with one or more metastasis located outside the vertebral bodies or pelvis, and/or visceral metastasis present [9, 38].

CYTOREDUCTIVE PROSTATE RADIOTHERAPY

Two randomised studies (STAMPEDE and HORRAD) have evaluated the role of cytoreductive local prostate radiotherapy in this cohort [38, 39].

The Systemic Therapy in Advancing Or Metastatic Prostate Cancer: Evaluation Of Drug Efficacy (STAMPEDE) collaborators explored the role of local prostate radiotherapy in 2,061 men with newly diagnosed metastatic prostate cancer receiving ADT, with 18% also receiving Docetaxel [38]. Although no OS advantage was demonstrated (HR 0.92, [95% CI 0.80-1.06]; p=0.27) in all burden metastatic disease, radiotherapy did improve failure-free survival (HR 0·76, [95% CI 0·68–0·84];

For peer review only - http://bmjopen.bmj.com/site/about/guidelines.xhtml

p<0·0001) [38]. Nevertheless, in the pre-specified subgroup of men with the CHAARTED definition of low burden disease, a significant OS was reported (3-year OS 81% vs 73%, HR 0.68, [95% CI 0.52–0.90]; p=0.007). As with any sub-group analyses this data needs it be interpreted cautiously. Furthermore, radiotherapy treatment (weekly or daily) had acceptable side-effects with only a 5% grade 3-4 adverse event rate [38].

The HORRAD phase three trial randomised 432 men to ADT with or without local prostate radiotherapy [39]. In accordance with STAMPEDE, no significant difference in OS was observed between the two groups (median 45 months in experimental arm versus 43 months with ADT alone; HR 0.90, [95% CI 0.70–1.14]; p = 0.40), though there was a non-significant trend towards improved overall survival in the 160 patients with low volume metastatic disease treated with radiotherapy (HR 0.68, 95% CI 0.42-1.10). This study however was criticised for its lack of pre-specified metastatic burden (including no knowledge of visceral disease) and potential underpowered sample size [40]. Both trials took place at a time when upfront systemic agents had not been fully introduced and thus the true "additive" effect of local prostate radiotherapy in a contemporary cohort remains unclear [2].

CYTOREDUCTIVE RADICAL PROSTATECTOMY

Historical data from the Southwest Oncology Group (SWOG) 8894 trial randomising 1,286 men with metastatic disease to bilateral orchidectomy with placebo or flutamide demonstrated, that a subgroup of men who underwent previous radical prostatectomy had a significantly reduced risk of death (HR 0.77, [95% CI 0.53–0.80]) [41]. Building on this, numerous retrospective series and registry data (e.g. Surveillance, Epidemiology, and End Results, SEER) have reported improved OS and CSS in men who undergo cytoreductive radical prostatectomy with low-burden or predominantly osseous disease [42-47].

At present, prospective evidence is limited to a 61-patient case-control study conducted by Heidenreich and colleagues [48]. Performed in men with <4 metastases and no visceral or extensive lymph node metastases, with a serum PSA level <1.0 ng/ml after neoadjuvant ADT, cytoreductive radical prostatectomy (CRP) and extended pelvic lymph node dissection (ePLND) led to a 12.1 month improvement in

For peer review only

For peer review only - http://bmjopen.bmj.com/site/about/guidelines.xhtml

PFS compared to the control arm ADT alone (38.6 months vs 26.5 months; $p = 0.0032$) [48]. There were no reported Grade 4 or 5 Clavien-Dindo classification complications within this study, confirming the surgical feasibility reported in previous retrospective studies [42, 43].

Multiple confirmatory randomised (NCT01751438 [BST]; NCT03655886 [LoMP II]) and single-arm studies (TRoMbone; NCT02716974; NCT03298087) are ongoing with, or without, MDT in this cohort [44, 49-52].

CYTOREDUCTIVE MINIMALLY INVASIVE ABLATIVE THERAPY (MIAT)

With regard to cytoreductive MIAT, a single retrospective study evaluating whole-gland cryotherapy in 23 men with a favourable response to 6-months ADT (PSA <1.0 ng/ml), </= cT3a disease, and limited bony metastasis, reported a 10-month survival advantage when compared to a matched cohort with ADT alone (35 months vs 25 months; HR 0.21, [95% CI 0.09–0.45]; $p = 0.0027$) [53].

Furthermore, the NCT02489357 pilot study interrogated the ‘cryo-immunological response’ with PD-1 blockade using the antibody (Pembrolizumab) in addition to cytoreductive cryotherapy [34]. In total 12 men with oligometastatic disease initiated 8-months of ADT and Pembrolizumab, with subsequent whole-gland cryotherapy [34]. Primary endpoint was PSA <0.6 ng/ml at 1 year, and this was met in 42% ($n = 5$). Median PFS was 14 months and median systemic therapy-free survival was 17.5 months [34]. There were no grade 3 adverse events, with Grade 1 (non-pad, occasional) urinary incontinence in 16.7% ($n = 2$) [34]. This profile is in keeping with the favourable early functional outcomes from cryotherapy in patients with non-metastatic disease [54]. With regard to safety there were no reported cases of rectal injury or fistulae in either study [34, 53]. In both studies men did not receive prior systemic therapy escalation and thus the ‘additive’ value of cytoreductive cryotherapy in such a cohort remains unclear [34, 53].

For peer review only

For peer review only - http://bmjopen.bmj.com/site/about/guidelines.xhtml

METASTASIS-DIRECTED THERAPY

In men with recurrent distant oligometastases, a number of early phase clinical trials (STOMP,ORIOLE, POPSTAR) have demonstrated promise with metastases-directed therapy (either SABR or metastatectomy), mainly with regards to improving ADT-free and early PFS [24, 55-57]. It is unclear the impact of such interventions on OS [24, 55, 56].

In *de novo* oligometastatic disease, a single pilot study including 20 men underwent sequential systemic therapy (ADT), surgery (cytoreductive radical prostatectomy + PLND +/- RPLND), and consolidation SABR to visible bone metastasis [58]. A novel endpoint of “undetectable PSA (</= 0.05 ng/ml) following testosterone recovery” was used and achieved in 20% (*n* = 4). However, 95% (*n* = 19) achieved an undetectable PSA, irrespective of testosterone suppression, after all three treatments. The addition of SABR accounted for an undetectable PSA in 21% (4/19) of this sub-group of men, when treatments were analysed separately [58].

METHODS AND ANALYSIS

STUDY DESIGN & DATES

IP2-ATLANTA is an unblinded, randomised, multicentre, interventional three-arm study with an active comparator arm incorporating standard of care (SOC) (**Figure 1**). Study participants will be randomised to: Control arm (standard of care); Intervention arm 1 (MIAT +/- pelvic lymph node dissection [PLND]) or Intervention arm 2 (prostate external beam radiotherapy [EBRT] +/- PLNRT OR cytoreductive radical prostatectomy [CRP] +/- PLND). Systemic therapy in all arms includes ADT +/- Docetaxel, Abiraterone Acetate, Enzalutamide, Apalutamide, as appropriate. Men with low-burden disease in intervention arms are eligible for metastases-directed therapy in the form of SABR or surgery (**Figure 1**).

Study recruitment commenced in April 2019, with planned embedded feasibility recruitment completed by February 2021, due to severe COVID-19 recruitment impact. Planned main phase II component recruitment and follow-up is expected to be completed by April 2022 and April 2024, respectively.

For peer review only - http://bmjopen.bmj.com/site/about/guidelines.xhtml

PATIENT AND PUBLIC INVOLVEMENT

A patient-involvement focus group was held with six patients who had advanced or metastatic prostate cancer, to determine initial patient acceptability and gauge important opinions on the proposed amendment and study design. Four patients had previously received radiotherapy as either their primary or secondary treatment. Comments from the group discussion were recorded along with anonymous questionnaires, which the patients returned, by post after the meeting. Two patient and public involvement (PPI) representatives were present during the HRA REC assessment. They will continue to be involved throughout the duration of the trial with the Trial Management Group and other patients not involved in the direct management of the study will be on the independent Trials Steering Committee.

STUDY POPULATION

Men who are willing to undergo local therapy to the prostate and selective metastases-directed therapy for newly diagnosed metastatic prostate cancer in addition to standard care systemic treatment upfront.

ELIGIBILITY

INCLUSION CRITERIA

1. Diagnosed with prostate cancer within 6 months of screening visit
2. Metastatic disease (Any T, Any N, M1+) of any grade, stage or Prostate Specific Antigen (PSA) level.
3. Fit to undergo standard of care systemic treatment for metastatic disease and both minimally invasive therapy and prostate EBRT/cytoreductive radical prostatectomy.
4. Performance status 0-2.
5. Histologically-proven local tumour.

EXCLUSION CRITERIA

1. Patient did not undergo and/or is unable to undergo standard of care baseline imaging tests for confirmation of metastatic status (CT abdomen/pelvis AND chest X-ray (or CT chest) AND radioisotope bone scan (or whole body imaging such as MRI or PET imaging as alternative to all preceding scans mentioned here) AND prostate MRI.

For peer review only - http://bmjopen.bmj.com/site/about/guidelines.xhtml

2. Prior exposure to long-term androgen deprivation therapy or hormonal therapy for the treatment of prostate cancer unless started within 4 months of screening visit.
3. Prior chemotherapy or local or systemic therapy for treatment of prostate cancer (apart from ADT or hormonal therapy as outlined above).

IDENTIFICATION OF PATIENTS & CONSENT

All men diagnosed with prostate cancer who go to a multidisciplinary team meeting or a tumour board as well as any man meeting the eligibility criteria prior to tumour board discussion will be identified for screening. Members of the tumour board will identify patients suitable for IP2-ATLANTA. The treating clinicians will mention the study and then the local research nurses/fellows, clinical trial coordinators, clinical trial practitioners or the treating clinicians will then approach the patient if they are interested. A Patient Information Sheet (PIS) will be given, or if agreed, emailed or posted out to the patient. Those patients already aware of the diagnosis can be approached by telephone to enquire as to their interest in the study so that a PIS can be then be sent out by email or post prior to a clinical visit. Patients will be given as much time as they need to read the PIS before consenting to participate (with a minimum of 24 hours).

RANDOMISATION

Stratified randomisation will take into account the following variables to create 16 strata in total:

- Intent to treat pelvic lymph nodes?: yes versus no
- Metastatic burden: low versus high (CHAARTED definition) [9].
- Intent to use systemic agent (i.e. Docetaxel, Abiraterone Acetate, Enzalutamide, Apalutamide)?: yes versus no
- Intent to use metastases directed therapy?: yes versus no

TRIAL TREATMENT

CONTROL ARM (STANDARD OF CARE)

Standard of care systemic treatment regimen is determined by the treating clinician and will be declared upfront prior to randomisation. The decision as to which SOC

For peer review only - http://bmjopen.bmj.com/site/about/guidelines.xhtml

systemic treatment is initiated should be made with reference to the current National Institute for Health and Care Excellence (NICE) and regional NHS clinically commissioned guidelines [59]. Presently, Docetaxel is recommended for use in all men with newly diagnosed metastatic prostate cancer who do not have significant co-morbidities [59, 60]. Alternatively, new anti-androgen compounds, including but not limited to, Abiraterone Acetate or Enzalutamide, are permitted if approved by regional NHS clinically commissioned guidelines [59, 61].

If radiotherapy is planned for local disease in men randomised to the SOC arm with low volume metastases, then this will be declared prior to randomisation by the treating clinician. For men with low burden disease, external beam prostate radiotherapy will be permitted and defined by the Local Radiotherapy Standard Operating Procedure (SOP) which reflects NHS clinically commissioned guidelines [62]. The use of PLNRT and/or MDT will not be permitted in the control arm. Palliative prostate radiotherapy for locoregional symptom control in men with high burden (>/=4) metastases will be permitted as per local clinical practice. Palliative bone radiotherapy for symptoms and prevention of fracture will be permitted in all men as per local clinical practice.

INTERVENTION ARMS

Whilst discussing the intervention arms we should also consider the impact that SOC systemic treatment may have on down-staging the local tumour. As the SOC systemic treatment would be administered prior to any local MIAT or CRP/EBRT in the intervention arms 1 and 2, an attempt at reclassifying the residual disease with a prostate MRI and biopsies would be pragmatic. This is to prevent patients from developing adverse events from unnecessary local treatment when they have no evidence of residual disease. Patients with positive post-SOC biopsies would then receive the local treatment as outlined below. Randomisation would occur at enrolment with planned intention to treat (ITT) and per-protocol analyses.

Biopsies at 6-9 months from initiation of SOC systemic therapy are part of the protocol during the embedded feasibility pilot. As we currently do not know the significance of residual disease after SOC systemic therapy, even if determined to be low-volume and low-grade, all patients with positive biopsies will be offered local treatment as per

For peer review only - http://bmjopen.bmj.com/site/about/guidelines.xhtml

randomisation. The pilot stage would obtain a point estimate of the magnitude of this response and also patient acceptability of a post-systemic therapy prostate biopsy [63-66]. Taken collectively this will assist in informing the study investigators of their ongoing utility in the main phase II component, where they are not presently mandated.

INTERVENTION ARM 1: MIAT

MIAT to the prostate with or without PLND in addition to SOC systemic treatment. The exact treatment protocol and modality used (cryotherapy or high-intensity focused ultrasound [HIFU]) will be set within the trial MIAT SOP. For those patients who are undergoing MIAT no local prostate radiotherapy will be given as part of the intervention. Radiotherapy can be administered for palliative reasons. PLND will be performed based on presence of resectable disease, patient fitness and consent/discussion with operating surgeon, as set out in the trials PLND with MIAT SOP. Cases may be referred for multidisciplinary discussion to the ATLANTA MIAT Quality Assurance Board.

INTERVENTION ARM 2: RADICAL THERAPY

In addition to SOC systemic treatment, radical therapy involves either: i) cytoreductive radical prostatectomy, with or without PLND, or ii) prostate EBRT, with or without simultaneous PLNRT. The actual modality will be based on physician and patient preference as well as patient co-morbidities and performance status.

The surgical technique is at the discretion and expertise of the surgical team but will reflect current UK surgical practice, laid down in the cytoreductive radical prostatectomy SOP. Trial surgeons must meet minimum case volume and optimal complication outcomes prior to operating in this trial. Further, they must receive peer-approval from the IP2-ATLANTA Surgeons Quality Assurance Board. For patients who are undergoing prostatectomy no local prostate radiotherapy will be given as part of the intervention. Radiotherapy can be given subsequently for palliative reasons.

Two local prostate radiotherapy dose and fractionation options are available:

For peer review only - http://bmjopen.bmj.com/site/about/guidelines.xhtml

- **60Gy in 20 fractions**. Treating the prostate to 60Gy and the seminal vesicles to 47Gy using a simultaneous integrated boost administered over 27 days. If the pelvic lymph nodes are to be treated then this will be done simultaneously to a dose of 47Gy in 20 fractions (if treated).
- **74-78Gy in 37-39 fractions**. Treat the prostate to 74-78Gy and the seminal vesicles to 60Gy, using as simultaneous integrated boost. If the pelvic lymph nodes are to be treated then this will done simultaneously to a dose of 55Gy in 37-39 fractions (in accordance to same fractions employed for treating the prostate and seminal vesicles).

The principles of pelvic nodal treatment within the study will follow those of the PIVOTALboost study (nodal arm-B), with variation to allow both dose and fractionation regimes [67]. Quality assurance for radiotherapy will be completed by the UK national Radiotherapy Trials Quality Assurance (RTTQA) team.

METASTASES-DIRECTED THERAPY: INTERVENTION ARMS 1 & 2

In men with low-burden disease in both intervention arms 1 and 2, MDT, may be used but intent-to-use MDT is to be declared prior to randomisation. In the case of a metastatic recurrence after MDT, a re-treatment with MDT would be allowed if there were new metastatic areas/locations. The imaging reporting of metastases as well as doses and protocol for MDT will be defined and determined by the Imaging Reporting SOP and Metastases-Directed Therapy SOP.

SABR should not be delivered whilst concurrent chemotherapy is being delivered. Concurrent hormonal therapy is acceptable. SABR delivered to metastases must be completed within 3 months of: prostate radiotherapy +/- PLNRT OR cytoreductive radical prostatectomy +/- PLND OR MIAT +/- PLND. Constraints on the dose and fractionations by anatomical site mirror all those defined in the SABR UK consortium guidelines v.6.1 guidelines (2019) or, if absent, in CORE Trial Radiotherapy, Planning & Delivery guidelines v.2.0 (2018) [68, 69]. Quality assurance for SABR will be completed by the RTTQA body.

For peer review only - http://bmjopen.bmj.com/site/about/guidelines.xhtml

STUDY ENDPOINTS & OUTCOME MEASURES

Primary and secondary endpoints for the study are presented in **Table 1**. Study outcome measures are presented in **Table 2**.

FOLLOW-UP

Follow-up will consist of 12-weekly serum PSA tests in the first year and 2-weekly thereafter, until mortality or 4 years after initial randomisation, whichever is first (**Table 3.**). PROMS will be collected every 6-months in the first year and annually thereafter, until mortality or 4 years after initial randomisation, whichever is first. Minimum follow-up for each patient will be 2 years. However, yearly follow-up will continue long term alongside linkage to national database.

PATIENT REPORTED OUTCOME MEASURES

The European Organisation for the Research and Treatment of Cancer (EORTC), Core Quality of Life Questionnaire-C30 (QLQ-C30), with prostate-specific, fatigue, elderly, general and bone metastases modules, International Prostatic Symptoms Score (IPSS), The Expanded Prostate Cancer Index Composite Bowel and Bladder (EPIC) and International Index of Erectile Function 5 (IIEF15) will be used. The EuroQol (EQ-5D-5L) will be used in the study as a generic measure of health-related quality-of-life which can be linked to public preferences. These data will be used to calculate quality-adjusted life-years as part of a future health economic evaluation. Patients agreeing to return questionnaires on quality-of-life will continue to complete quality of life data for 4 years after enrolment.

STUDY VISITS

Follow up visits for the administration of SOC therapy and clinical review will occur in accordance with the local hospitals follow-up protocols. In the intervention arms, of the embedded feasibility pilot only, a prostate MRI, systematic and targeted transrectal or transperineal biopsy will be performed at 6-9 months following the initiation of the SOC therapy. In the main phase II component, these procedures can be carried out by local centres at the discretion of local clinicians and when they are, data should be collected on their outcomes.

For peer review only - http://bmjopen.bmj.com/site/about/guidelines.xhtml

For those randomised to MIAT or CRP/EBRT, a date for treatment(s) will be booked in accordance with the local hospital waiting lists. Removal of the urethral catheter after MIAT or CRP will occur after a minimum period of 7-days during a hospital visit or can be removed either at the GP surgery or at a local hospital to the patient.

Further clinical reviews will occur as SOC visits at 12, 26, 28, 32, 34 and 52 weeks in the 1st year and 24-weekly intervals thereafter until mortality or 2 years after enrolment, whichever is first.

Further Follow-up Imaging

Follow-up imaging to determine response from treatment on primary and metastatic disease will not be protocolled but we recommend imaging to take place when there is suspicion of progression, such as patients with a rising PSA (i.e. biochemical failure). The appropriate imaging will be chosen as per the local hospital resources and policies. We envisage that the majority will perform a combination of a prostate MRI, bone scintigraphy, PET-CT/MRI, whole body MRI or CT chest/abdomen/pelvis.

Long-term outcomes

Patients will be consented for their details to be linked to national registries for survival information such as NHS Information Centre/Office of National Statistics (ONS) in England/Wales and General Register Office in Scotland, Hospital Episode Statistics (HES). This ONS-HES linkage however is an optional consent.

STATISTICAL ANALYSES AND SAMPLE SIZE CALCULATION

Embedded Feasibility and Pilot

We are seeking to determine whether the randomisation of men with metastatic disease is feasible and whether men are compliant to the therapy following randomisation. We aim to approach 80 patients from up to 17 centres in the UK over a 6-month period to allow us to estimate a 33% recruitment rate with 95% confidence interval width of approximately ± 10 percentage points.

For peer review only - http://bmjopen.bmj.com/site/about/guidelines.xhtml

Main phase II component

The study will have 80% power to detect a treatment difference with a hazard ratio 0.7 in favour of any of the intervention arms compared to the control at a two-sided 5.0% significance level. This is based on the assumption that the accrual period will be uniform over 24 months, that the follow-up period will be 24 months, and that the median progression-free survival is 37 months. This calculation may be adjusted depending on the compliance rate assessed during the feasibility stage. The overall sample size will be 918 participants considering a 5% loss to follow-up (291 participants per group, 873 participants for three arms). This will allow the detection of an effect size of 9.2% increase in progression free survival at 24 months.

ADVERSE EVENT REPORTING

The Common Terminology Criteria for Adverse Events (CTCAEv5.0) domain will be used to report adverse events [70].

DATA COLLECTION

The principal means of data collection from participant visits will be Electronic Data Capture (EDC) using the web-based InForm database. All study data will be entered into electronic Case Report Forms (eCRFs) in a database provided by the Sponsor. All eCRFs will be completed using de-identified data.

DATA MONITORING & ARCHIVING

A combined independent data monitoring and trial steering committee will meet twice a year. All trial documentation, including that held at participating sites and the trial coordinating centre, will be archived for a minimum of 10 years following the end of the study.

ETHICS AND DISSEMINATION

This trial was approved by the Health Research Authority (HRA) Research Ethics Committee Wales (REC5; 19/WA0005). The results will be submitted for publication in peer-reviewed journals and submitted to the REC within a year of the end of the study.

For peer review only

For peer review only - http://bmjopen.bmj.com/site/about/guidelines.xhtml

TRIAL FUNDING, ORGANISATION AND ADMINISTRATION

IP2-ATLANTA trial was approved by the HRA Wales REC 5 (19/WA0005). IP2-ATLANTA is funded by the Wellcome Trust (204998/Z/16/Z). The study will be monitored periodically by trial monitors to assess the progress of the study, verify adherence to the protocol, ICH GCP E6 guidelines and other national/international requirements and to review the completeness, accuracy and consistency of the data

DISCUSSION

IP2-ATLANTA is a multicentre, phase II, randomised controlled trial. The study will provide Level I evidence on oncological outcomes from prostate minimally invasive ablative therapy or radical therapy, in combination with metastasis-directed therapy, against standard of care treatment alone, in men with newly-diagnosed hormone sensitive metastatic prostate cancer. If either intervention arm is proven to provide significant oncological benefit this will have wide-reaching implications on the current standard of care paradigm.

CONCLUSION

IP2-ATLANTA addresses an important research gap in the role of sequential systemic, local cytoreductive and metastasis-directed interventions in men with newly-diagnosed metastatic prostate cancer.

TRIAL STATUS

IP2-ATLANTA is open to recruitment in 13 centres in England and Wales and expected to complete its embedded feasibility pilot phase by late 2020 [71].

Acknowledgements: We would like to thank all the participants, study PI, trial clinicians, research nurses, Imperial Clinical Trial Unit staff and other site staff who have been responsible for setting up, recruiting participants and collecting the data for the IP2-ATLANTA trial. Further we are grateful for the ongoing support of the Trial Management Group and our trial patient representative. Finally, we would like to thank the trial oversight provided by ICTU and our trial funder the Wellcome Trust.

For peer review only - http://bmjopen.bmj.com/site/about/guidelines.xhtml

Contributors: Conception and design of ATLANTA trial: HUA, MJC, MW, TTS, TD, AF, VK, MG, NS, JS, ED, FF, NKN, ME. All authors have read and approved the final manuscript: MJC, TTS, KS, ED, JS, FF, NS, MG, AF, NKN, ME, OFN, KTJ, DP, SG, DB, GH, JM, DS, MK, AI, CB, RAP, NA, CH, IS, BR, GH, SM, BK, SM, VK, TD, JNS, MW, HUA.

Funding: The trial was funded by the Wellcome Trust (204998/Z/16/Z)

Patient consent for publication: Not required.

Conflicts of Interest

Martin J. Connor's research is support by University College London Hospitals (UCLH) Charity and the Wellcome Trust.

Kamalram Thippu Jayaprakash is currently supported by a research grant from the UK National Institute of Health Research (NIHR) Clinical Research Network Eastern. He has received educational/ and travel grants from Bayer UK, Janssen Oncology, Pfizer; Roche, Takeda.

Hashim U. Ahmed's research is supported by core funding from the United Kingdom's National Institute of Health Research (NIHR) Imperial Biomedical Research Centre. Ahmed currently receives funding from the Wellcome Trust, Prostate Cancer UK, MRC (UK), Cancer Research UK, Sonacare Inc., Trod Medical, and Sophiris Biocorp for trials in prostate cancer. Ahmed was a paid medical consultant for Sophiris Biocorp, Sonacare Inc. and BTG in the past 3 years.

All other authors have no declaration

For peer review only

References

1 Smittenaar CR, Petersen KA, Stewart K, et al. Cancer incidence and mortality projections in the UK until 2035, *Br J Cancer* 2016;115:1147-55 doi:10.1038/bjc.2016.304 [doi] [published Online First: October 25].

2 Connor MJ, Shah TT, Horan G, et al. Cytoreductive treatment strategies for de novo metastatic prostate cancer, *Nature Reviews Clinical Oncology* 2019:1-15.

3 James ND, Spears MR, Clarke NW, et al. Survival with newly diagnosed metastatic prostate cancer in the “docetaxel era”: data from 917 patients in the control arm of the STAMPEDE trial (MRC PR08, CRUK/06/019), *Eur Urol* 2015;67:1028-38.

4 Miyake H, Matsushita Y, Watanabe H, et al. Prognostic significance of time to castration resistance in patients with metastatic castration-sensitive prostate cancer, *Anticancer Res* 2019;39:1391-6.

5 Chi KN, Agarwal N, Bjartell A, et al. Apalutamide for metastatic, castration-sensitive prostate cancer. *N Engl J Med* 2019;381:13-24.

6 Davis ID, Martin AJ, Stockler MR, et al. Enzalutamide with standard first-line therapy in metastatic prostate cancer. *N Engl J Med* 2019;381:121-31.

7 James ND, Sydes MR, Clarke NW, et al. Addition of docetaxel, zoledronic acid, or both to first-line long-term hormone therapy in prostate cancer (STAMPEDE): survival results from an adaptive, multiarm, multistage, platform randomised controlled trial, *Lancet* 2016;387:1163-77 doi:10.1016/S0140-6736(15)01037-5 [doi] [published Online First: March 19].

8 Fizazi K, Tran N, Fein L, et al. Abiraterone plus prednisone in metastatic, castration-sensitive prostate cancer, *N Engl J Med* 2017;377:352-60.

9 Sweeney CJ, Chen Y, Carducci M, et al. Chemohormonal therapy in metastatic hormone-sensitive prostate cancer, *N Engl J Med* 2015;373:737-46.

10 Weichselbaum RR, Hellman S. Oligometastases revisited, *Nature reviews Clinical oncology* 2011;8:378.

11 Connor MJ, Winkler M, Ahmed HU. Survival in Oligometastatic Prostate Cancerâ€"A New Dawn or the Will Rogers Phenomenon? *JAMA oncology* 2020;6:185-6.

12 Sita-Lumsden A, Dart DA, Waxman J, et al. Circulating microRNAs as potential new biomarkers for prostate cancer, *Br J Cancer* 2013;108:1925.

13 Selth LA, Townley SL, Bert AG, et al. Circulating microRNAs predict biochemical recurrence in prostate cancer patients, *Br J Cancer* 2013;109:641.

14 Alix-Panabières C, Riethdorf S, Pantel K. Circulating tumor cells and bone marrow micrometastasis, *Clinical Cancer Research* 2008;14:5013-21.

15 Lorente D, Olmos D, Mateo J, et al. Decline in circulating tumor cell count and treatment outcome in advanced prostate cancer, *Eur Urol* 2016;70:985-92.

16 Lilleby W, Stensvold A, Mills IG, et al. Disseminated tumor cells and their prognostic significance in nonmetastatic prostate cancer patients, *International journal of cancer* 2013;133:149-55.

17 Morgan TM, Lange PH, Porter MP, et al. Disseminated tumor cells in prostate cancer patients after radical prostatectomy and without evidence of disease predicts biochemical recurrence, *Clinical cancer research* 2009;15:677-83.

For peer review only

For peer review only - http://bmjopen.bmj.com/site/about/guidelines.xhtml

18 Weckermann D, Polzer B, Ragg T, et al. Perioperative activation of disseminated tumor cells in bone marrow of patients with prostate cancer, *J Clin Oncol* 2009;27:1549-56.

19 Comen E, Norton L, Massague J. Clinical implications of cancer self-seeding, *Nature reviews Clinical oncology* 2011;8:369.

20 Kim M, Oskarsson T, Acharyya S, et al. Tumor self-seeding by circulating cancer cells, *Cell* 2009;139:1315-26.

21 Shiozawa Y, Pedersen EA, Havens AM, et al. Human prostate cancer metastases target the hematopoietic stem cell niche to establish footholds in mouse bone marrow, *J Clin Invest* 2011;121:1298-312.

22 Sleeman JP. The metastatic niche and stromal progression, *Cancer Metastasis Rev* 2012;31:429-40.

23 Gundem G, Van Loo P, Kremeyer B, et al. The evolutionary history of lethal metastatic prostate cancer, *Nature* 2015;520:353.

24 Ryan Phillips, William Yue Shi, Matthew Deek, et al. Outcomes of Observation vs Stereotactic Ablative Radiation for Oligometastatic Prostate Cancer. The ORIOLE Phase 2 Randomized Clinical Trial. *JAMA Oncology* 2020 [published Online First: Mar 26,].

25 Palma DA, Olson RA, Harrow S, et al. Stereotactic ablative radiation therapy for the comprehensive treatment of oligometastatic tumors (SABR-COMET): results of a randomized trial, *International Journal of Radiation Oncology• Biology• Physics* 2018;102:S3-4.

26 Golden EB, Demaria S, Schiff PB, et al. An abscopal response to radiation and ipilimumab in a patient with metastatic non–small cell lung cancer, *Cancer immunology research* 2013;1:365-72.

27 Strigari L, Mancuso M, Ubertini V, et al. Abscopal effect of radiation therapy: Interplay between radiation dose and p53 status, *Int J Radiat Biol* 2014;90:248-55.

28 Postow MA, Callahan MK, Barker CA, et al. Immunologic correlates of the abscopal effect in a patient with melanoma, *N Engl J Med* 2012;366:925-31.

29 Brooks ED, Chang JY. Time to abandon single-site irradiation for inducing abscopal effects, *Nature Reviews Clinical Oncology* 2018:1.

30 Kubo M, Satoh T, Ishiyama H, et al. Enhanced activated T cell subsets in prostate cancer patients receiving iodine-125 low-dose-rate prostate brachytherapy, *Oncol Rep* 2018;39:417-24.

31 Sabel MS. Cryo-immunology: a review of the literature and proposed mechanisms for stimulatory versus suppressive immune responses, *Cryobiology* 2009;58:1-11.

For peer review only

For peer review only - http://bmjopen.bmj.com/site/about/guidelines.xhtml

32 Ablin RJ, Soanes WA, Gonder MJ. Prospects for cryo-immunotherapy in cases of metastasizing carcinoma of the prostate, *Cryobiology* 1971;8:271-9.

33 Benzon B, Glavaris SA, Simons BW, et al. Combining immune check-point blockade and cryoablation in an immunocompetent hormone sensitive murine model of prostate cancer, *Prostate cancer and prostatic diseases* 2018;21:126-36.

34 Ross AE, Hurley PJ, Tran PT, et al. A pilot trial of pembrolizumab plus prostatic cryotherapy for men with newly diagnosed oligometastatic hormone-sensitive prostate cancer, *Prostate cancer and prostatic diseases* 2020;23:184-93.

35 Connor MJ, Winkler M, Ahmed HU. Cytoreductive cryotherapy for newly diagnosed oligometastatic hormone-sensitive prostate cancer, *Prostate Cancer and Prostatic Diseases* 2020:1-2.

36 Tosoian JJ, Gorin MA, Ross AE, et al. Oligometastatic prostate cancer: definitions, clinical outcomes, and treatment considerations, *Nature Reviews Urology* 2017;14:15.

37 Khoo V. New concepts in prostate cancer management: the conundrum of managing oligometastatic disease in prostate cancer—through the looking glass darkly, *Clin Radiol* 2019.

38 Parker CC, James ND, Brawley CD, et al. Radiotherapy to the primary tumour for newly diagnosed, metastatic prostate cancer (STAMPEDE): a randomised controlled phase 3 trial, *Lancet* 2018;392:2353-66 doi:S0140-6736(18)32486-3 [pii] [published Online First: December 01].

39 Boevé LM, Hulshof MC, Vis AN, et al. Effect on survival of androgen deprivation therapy alone compared to androgen deprivation therapy combined with concurrent radiation therapy to the prostate in patients with primary bone metastatic prostate cancer in a prospective randomised clinical trial: data from the HORRAD trial, *Eur Urol* 2019;75:410-8.

40 Gregucci F, Fiorentino A. Re: Liselotte MS Boevé, Maarten CCM Hulshof, André N. Vis, et al. Effect on Survival of Androgen Deprivation Therapy Alone Compared to Androgen Deprivation Therapy Combined with Concurrent Radiation Therapy to the Prostate in Patients with Primary Bone Metastatic Prostate Cancer in a Prospective Randomized Clinical Trial: Data from the HORRAD Trial. Eur Urol 2019; 75: 410-8. *Eur Urol* 2019;75:e129.

41 Thompson IM, Tangen C, Basler J, et al. Impact of previous local treatment for prostate cancer on subsequent metastatic disease, *J Urol* 2002;168:1008-12.

42 Jang WS, Kim MS, Jeong WS, et al. Does robot-assisted radical prostatectomy benefit patients with prostate cancer and bone oligometastases? *BJU Int* 2018;121:225-31.

43 Sooriakumaran P, Karnes J, Stief C, et al. A multi-institutional analysis of perioperative outcomes in 106 men who underwent radical prostatectomy for distant metastatic prostate cancer at presentation, *Eur Urol* 2016;69:788-94.

For peer review only

For peer review only - http://bmjopen.bmj.com/site/about/guidelines.xhtml

44 Sooriakumaran P. Testing radical prostatectomy in men with prostate cancer and oligometastases to the bone: a randomized controlled feasibility trial, *BJU Int* 2017;120:E8-E20.

45 Heidenreich A, Fossati N, Pfister D, et al. Cytoreductive radical prostatectomy in men with prostate cancer and skeletal metastases, *European urology oncology* 2018;1:46-53.

46 Culp SH, Schellhammer PF, Williams MB. Might men diagnosed with metastatic prostate cancer benefit from definitive treatment of the primary tumor? A SEER-based study, *Eur Urol* 2014;65:1058-66 doi:10.1016/j.eururo.2013.11.012 [doi] [published Online First: June 01].

47 Rusthoven CG, Jones BL, Flaig TW, et al. Improved survival with prostate radiation in addition to androgen deprivation therapy for men with newly diagnosed metastatic prostate cancer, *Journal of Clinical Oncology* 2016;34:2835-42.

48 Heidenreich A, Pfister D, Porres D. Cytoreductive radical prostatectomy in patients with prostate cancer and low volume skeletal metastases: results of a feasibility and case-control study, *J Urol* 2015;193:832-8.

49 Pienta K. A Study of Definitive Therapy to Treat Prostate Cancer (oligo-mets) NCT02716974 . ;2019.

50 Nickols N, Rettig M. NCT03298087 Systemic and Tumor-Directed Therapy for Oligometastatic Prostate Cancer.

51 Chapin BF, Mcguire SE, Wang X, et al. No title, *A prospective, multicenter, randomized phase II trial of best systemic therapy (BST) or BST plus definitive treatment (Surgery or Radiation) of the primary tumor in metastatic prostate cancer.* 2015.

52 Nicolaas L. NCT03655886. Testing radical prostatectomy in men with prostate cancer and oligometastases to the bone: a randomized controlled feasibility trial (LoMP II). 2018.

53 Sheng M, Wan L, Liu C, et al. Cytoreductive cryosurgery in patients with bone metastatic prostate cancer: A retrospective analysis, *Kaohsiung J Med Sci* 2017;33:609-15.

54 Shah TT, Peters M, Eldred-Evans D, et al. Early-Medium-Term Outcomes of Primary Focal Cryotherapy to Treat Nonmetastatic Clinically Significant Prostate Cancer from a Prospective Multicentre Registry, *Eur Urol* 2019.

55 Siva S, Bressel M, Murphy DG, et al. Stereotactic abative body radiotherapy (SABR) for oligometastatic prostate cancer: a prospective clinical trial, *Eur Urol* 2018;74:455-62.

For peer review only

56 Ost P, Reynders D, Decaestecker K, et al. Surveillance or metastasis-directed therapy for oligometastatic prostate cancer recurrence: a prospective, randomized, multicenter phase II trial, *Journal of Clinical Oncology* 2017;36:446-53.

57 Connor MJ, Smith A, Miah S, et al. Targeting oligometastasis with stereotactic ablative radiation therapy or surgery in metastatic hormone-sensitive prostate cancer: a systematic review of prospective clinical trials, *European Urology Oncology* 2020.

58 O'Shaughnessy MJ, McBride SM, Vargas HA, et al. A pilot study of a multimodal treatment paradigm to accelerate drug evaluations in early-stage metastatic prostate cancer, *Urology* 2017;102:164-72.

59 NICE, Guideline Updates Team UK. Prostate cancer: diagnosis and management, 2019.

60 National Institute for Health and Care Excellence. Prostate cancer: diagnosis and management [B] Evidence review for docetaxel in people with hormone sensitive prostate cancer [NG131] . 2019.

61 Scottish Medicines Consortium. Abiraterone acetate with prednisone or prednisolone for the treatment of newly diagnosed high risk metastatic hormone sensitive prostate cancer in adult men in combination with androgen deprivation therapy [SMC2215]. 2020.

62 NHS England Clinical Commissioning. External beam radiotherapy for patients presenting with hormone sensitive, low volume metastatic prostate cancer at the time of diagnosis [P200802P] (URN: 1901) . 2020.

63 Köllermann J, Caprano J, Budde A, et al. Follow-up of nondetectable prostate carcinoma (pT0) after prolonged PSA-monitored neoadjuvant hormonal therapy followed by radical prostatectomy, *Urology* 2003;62:476-80.

64 Bream MJ, Dahmoush L, Brown JA. pT0 Prostate Cancer: predictive clinicopathologic features in an American population, *Current urology* 2013;7:14.

65 McKay RR, Ye H, Xie W, et al. Evaluation of intense androgen deprivation before prostatectomy: a randomized phase II trial of enzalutamide and leuprolide with or without abiraterone, *Journal of Clinical Oncology* 2019;37:923.

66 Evans AJ. Treatment effects in prostate cancer, *Modern Pathology* 2018;31:110-21.

67 Syndikus I. ISRCTN80146950. A phase III randomised controlled trial of prostate and pelvis versus prostate alone radiotherapy with or without prostate boost (PIVOTALBoost). 2019 [published Online First: March,].

68 Khoo V. Conventional Care Versus Radioablation (Stereotactic Body Radiotherapy) for Extracranial Oligometastases (CORE) NCT02759783. ;2019.

For peer review only

852 69 The Faculty of Clinical Oncology of The Royal College of Radiologists. Stereotactic
853 Ablative Body
854 Radiation Therapy (SABR):
855 A Resource. Version 6.1. 2019.

856 70 U.S. DEPARTMENT OF HEALTH AND HUMAN SERVICES. Common
857 Terminology Criteria
858 for Adverse Events (CTCAE) Version 5.0. 2017.

859 71 Connor MJ, Shah TT, Sukumar J, et al. Initial experience of the adjuvant treatments
860 to the local tumor for metastatic prostate cancer: Assessment of novel treatment
861 algorithms, a multicenter, phase II randomized controlled trial (IP2-ATLANTA). *Journal*
862 *of Clinical Oncology* 2020.

863
864

For peer review only

For peer review only - http://bmjopen.bmj.com/site/about/guidelines.xhtml

865 ## Table & Figure Legends

867 **Table 1**. Study primary and secondary endpoints

869 **Table 2**. Study outcome measures

871 **Table 3.** Study visit schedule. *Legend: [1]Randomisation should be performed within 7 days from screening visit*

874 **Figure 1**. Study Flowchart. *Legend: MIAT – Minimally invasive ablative therapy; CRP – Cytoreductive Radical Prostatectomy; PLND – Pelvic Lymph Node Dissection; PLNRT – Pelvic Lymph Node Radiotherapy; EBRT – External Beam Radiotherapy. *Systemic therapy is not limited to listed agents, *Prostate only EBRT may be perform in selected men with low-burden disease, if declared prior to randomisation and local SOC.*

For peer review only

Table 1. Study primary and secondary endpoints

**PRIMARY ENDPOINT: Embedded Feasibility Pilot**	a) Recruitment, randomisation and compliance to allocation<br>b) Adverse events<br>c) Proportion of patients with pathological complete response on post systemic therapy prostate biopsy at 6-9 months.
**PRIMARY ENDPOINT: Phase II**	a) Progression-free survival (PFS)<br>*Defined as a composite outcome of biochemical failure; local progression; lymph node progression or bone metastases progression (new sites); or progression or development of new distant metastases, defined as lymph nodes outside the pelvis, bone or organ involvement or skeletal-related events confirmed as progression as in the STAMPEDE randomised study (**Assessment of Progression**; **Supplementary Material**) [42].*
**SECONDARY ENDPOINT: Phase II**	a) Adverse events and side-effect profile<br>b) Predictive factors for PFS and OS in each arm.<br>c) Effect on PFS or OS from varying radiotherapy dosage and schedules<br>d) Effect on PFS and OS stratified by volume and site for local and metastatic disease<br>e) Effect on PFS and OS stratified by the use of metastases directed therapy.<br>f) Effect on PFS using an alternative definition of failure, defined as a PSA increase of >/=25% and >/=2ng/mL if PSA was >/=2ng/mL from baseline, or a PSA increase of >/=25% if PSA was <2ng/mL at random assignment.<br>g) Effect on PFS using an alternative definition of local progression of a soft tissue metastatic lesion: defined as an increase of >/=20% in the largest tumour dimension with a minimum absolute increase of 5mm. Local progression of bone metastases to be assessed using MD Anderson Cancer Center–criteria with a >/=25% increase in the size of a measurable lesion on CT or a >/=25% increase in the size of ill-defined lesions on CT considered to be progression (1, 2).<br>h) Costs and resource utilisation for future cost-effectiveness analyses<br>i) In those men undergoing repeat biopsies after 6-9 months of standard of care systemic therapy, the proportion of patients with negative biopsies.<br>j) In those men undergoing repeat prostate/pelvic MRI after 6-9 months of standard of care systemic therapy, the proportion of patients with a negative prostate MRI for local tumour.<br>k) In those men undergoing repeat imaging (local prostate/pelvic and / or other body areas) after 6-9 months of standard of care systemic

For peer review only

	therapy, the proportion of patients with reduction on imaging of metastatic tumour deposits.

For peer review only

For peer review only - http://bmjopen.bmj.com/site/about/guidelines.xhtml

Table 2. Study outcome measures

**PRIMARY OUTCOMES: Embedded Feasibility Pilot**	a) Compliance to randomised arm<br>b) Recruitment and randomisation rate<br>c) Safety (adverse events)<br>d) Proportion of patients with complete pathological response on post SOC systemic therapy prostate biopsies at 6-9 months.
**PRIMARY OUTCOMES: Phase II**	a) Progression-free survival (PFS)
**SECONDARY OUTCOMES: Phase II**	a) Urinary, sexual and rectal side-effects<br>b) Patient-reported outcomes using validated questionnaires<br>c) Progression on PSA and imaging and impact of clinical features on progression<br>d) Health-related quality-of-life<br>e) Data on costs and resource utilisation for future cost-effectiveness analysis

For peer review only

Table 3. Study visit schedule

	Screening	Treatment					Post-treatment	Follow-up
**Visit**	**1**	**2**	**3**	**4**	**5**	**6**	**7**	**8 onwards**
Week (+/-)	0	12 (+/- 4)	26 (+/-12)	28 (+/- 12)	32 (+/- 12)	34 (+/- 12)	52 (+/- 4)	Every 24 weeks (+/- 4) (year 2-4)
Informed consent	X							
Inclusion & exclusion criteria	X							
Demography	X							
Medical history	X							
Vital signs/physical examination/clinical or subject assessment	X		X				As deemed necessary based on medical history and AE review	
PSA Blood Test	X	X	X			X	X	X
PROMS Questionnaires	X		X				X	X (at 24 months only)
Review/reporting of patient AEs/SAEs (may be performed via a face to face or telephone or email consultation)		X	X	X	X	X	X	X
Blood and urine tests including those for biobanking	X		X			X	At time of failure or at 24, 36, 48 months	
Randomisation	X[1]							
Standard of Care Therapy	X	X	X	X	X	X	X	X
Imaging Tests (Combination of but not limited to CT, X-rays, PET, MRI, Bone Scan)	If not already performed (SOC)		Recommended but not protocolled				As deemed necessary	
Prostate MRI	If not already performed (SOC)		X Mandatory in Pilot only. During Main Phase can be conducted at clinician discretion within standard of care process				As deemed necessary	
Biopsy			X Mandatory in Pilot only. During Main Phase can be conducted at clinician discretion within standard of care process				As deemed necessary	
Testosterone blood Test	X (if available)		X (recommended but not mandated)				As deemed necessary	
Treatment in intervention arms					X			
Removal of catheter						X		

Legend: [1]Randomisation should be performed within 7 days from screening visit

For peer review only - http://bmjopen.bmj.com/site/about/guidelines.xhtml

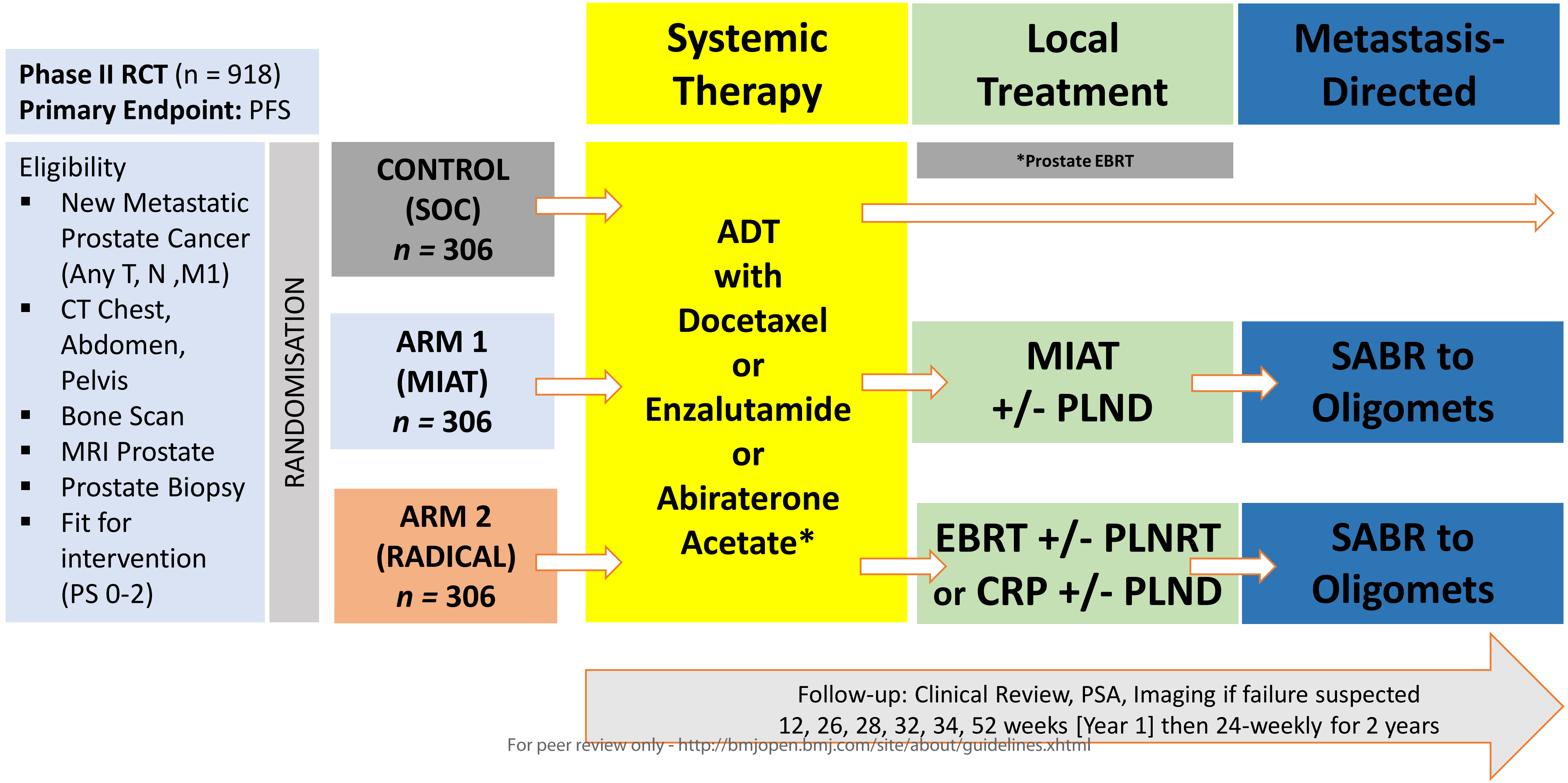

For peer review only - http://bmjopen.bmj.com/site/about/guidelines.xhtml

Supplementary Material

1.0 Assessment of progression

Baseline radiological examinations will be performed prior to enrolment. In the pilot only, prostate MRI and biopsy will be repeated at 26 (+/-12) weeks. In the main phase it is not protocol-mandated, that all patients have imaging scans repeated at the same time point (26 weeks +/- 12 weeks) and whenever clinically appropriate, such as in those with a low PSA value at enrolment or when there are concerns for progression i.e. biochemical failure, new bone pain, Skeletal Related Event (SRE). Clinicians can, at their own discretion, conduct a repeat MRI and biopsy of the prostate during the main phase.

The following outcomes should be reported:

- Biochemical failure
- Local progression
- Lymph node progression
- Bone metastases progression (new sites)
- Progression or development of new distant metastases, defined as lymph nodes outside the pelvis, bone or organ involvement
- Skeletal-related events confirmed as progression (see below).

Biochemical Failure

For the purposes of this trial, a unique threshold PSA value for biochemical failure is calculated, referred to as the PSA progression value. This value is derived for each patient based on their PSA nadir**,** defined as the lowest PSA value reported between randomisation and 6 months in the trial. The exact method for deriving the progression value for a patient depends on the value of their PSA nadir, and how this compares to their pre-treatment PSA value (i.e. the extent of the fall in PSA from the starting point).

The PSA progression value is calculated in one of three ways:

1. If the lowest recorded PSA value in the 26 weeks following randomisation is more than 4ng/ml and more than 50% of the pre-treatment PSA level then the patient fulfils the criteria for immediate treatment failure.
2. For patients whose PSA nadir in the 26 weeks following randomisation is less than or equal to 50% of the pre-treatment PSA level but remains above 4ng/ml, biochemical failure will be defined as a rise of 50% above the nadir level.
3. For patients whose PSA nadir is less than or equal to 4ng/ml, biochemical failure is defined as at least a 50% rise above the nadir value that is also above 4ng/ml.

Confirming biochemical failure: The timing and thus assessment of PSA needs to be considered because rises in PSA can occur due to non-cancer related causes such as after procedures, biopsies or urinary tract infection (UTI's). Confirmatory samples are needed in all cases of a rising PSA, prior to assigning an outcome of biochemical failure. After biochemical failure is confirmed for the first time it need not be reported again.

In the case that the raised PSA value reaches the progression value, a confirmatory PSA test should be performed after at least 1 week or 4 weeks after the completion of treatment in cases of UTI's, procedures or biopsies. Biochemical failure is confirmed

For peer review only

if the second value is around the same level or higher. The date of PSA progression should be provided as the date of the **first** raised PSA that fulfilled the definition for progression.

A confirmatory PSA is not required if there are other signs of progression e.g. progression of cancer related symptoms (clinical progression) or new radiological progression.

Second line treatment commenced specifically for biochemical failure should not start until the trial definition for biochemical failure has been met. However, if second line treatment does start before the trial definition is met then report the closest PSA value prior to the treatment start date as the progression value. This is not required if second line treatment is being started for other signs of progression e.g. clinical or radiological.

Testosterone levels: are required when reporting biochemical progression whilst receiving hormone treatment to confirm the diagnosis of castrate resistant prostate cancer.

Local, Lymph Node and Metastatic Failure
For each of local, lymph node and distant metastases progression, **both** the following should be reported:

- Date of first clinical/symptomatic progression
- Date of first objective/radiological progression.

Skeletal-related Events
Skeletal-related events (SREs) are defined as:

- Pathological fracture
- Spinal cord compression
- Requirement for RT to bone (e.g. for pain or impending fracture)
- Requirement for surgery (e.g. for prevention or management of fracture).

All SREs should be investigated further to establish whether or not the patient has progressed and only logged as progression if confirmed clinically or on imaging to be due to metastatic prostate cancer.

For peer review only

2.0 Study Management

All trial documentation, including that held at participating sites and the trial coordinating centre, will be archived for a minimum of 10 years following the end of the study. Subject files and other source data (including copies of protocols, CRFs, original reports of test results, correspondence, records of informed consent, and other documents pertaining to the conduct of the study) must be retained. Documents should be stored in such a way that they can be accessed/data retrieved later. Consideration should be given to security and environmental risks.

No study document will be destroyed without prior written agreement between the Sponsor and the investigator. Should the investigator wish to assign the study records to another party or move them to another location, written agreement must be obtained from the Sponsor.

Storage and handling of confidential trial data and documents will be in accordance with the Data Protection Act 2018 (UK).

Study Management Structure

A Trial Steering Committee (TSC) will be convened including as a minimum an Independent Chair, Independent Clinician, the Chief Investigator and Study Manager. The role of the TSC is to provide overall supervision of trial conduct and progress. Details of membership, responsibilities and frequency of meetings will be defined in a separate Charter.

A Trial Management Group (TMG) will be convened including the Chief Investigator, co-investigators and key collaborators, Study Statistician and Study Manager. The TMG will be responsible for day-to-day conduct of the trial and operational issues. Details of membership, responsibilities and frequency of meetings will be defined in separate Terms of Reference. When necessary, decisions will be referred to the TSC. Meetings will be scheduled in a risk-adapted manner to allow for the review of events during the trial.

A combined data monitoring and trial steering committee will meet twice a year basis. The composition of this committee will include but not be limited to the Chief Investigator, Trial Statistician, Trial Coordinator, Trials Unit representative, Research nurse and Patient representative

In case of early discontinuation of the study, the Follow-up Visit assessments should be performed for each subject, as far as possible.

The following reasons may result in early discontinuation:

- Early evidence that a treatment arm is harmful. If only one treatment arm is deemed to be harmful then the remaining arms of the study may continue as planned,

OR

- It is not feasible to reach the planned outcomes (A hazard ration of 0.7 to 1 will make the intervention not worth progressing with given the severe adverse effects associated with it)

For peer review only

The statistical criteria for termination of the study will be detailed in the statistical analysis plan (SAP).

A study-specific risk assessment will be performed prior to the start of the study to assign a risk category of 'low', 'medium' or 'high' to the trial. Risk assessment will be carried out by the ICTU QA Manager in collaboration with the Study Manager and the result will be used to guide the monitoring plan. The risk assessment will consider all aspects of the study and will be updated as required during the course of the study. The study will be monitored periodically by trial monitors to assess the progress of the study, verify adherence to the protocol, ICH GCP E6 guidelines and other national/international requirements and to review the completeness, accuracy and consistency of the data.

Monitoring procedures and requirements will be documented in a Monitoring Plan, developed in accordance with the risk assessment.

Quality Control will be performed according to ICTU/ internal procedures. The study may be audited by a Quality Assurance representative of the Sponsor and/or ICTU. All necessary data and documents will be made available for inspection.

The study may be subject to inspection and audit by regulatory bodies to ensure adherence to GCP and the NHS Research Governance Framework for Health and Social Care (2nd Edition).

Dissemination of findings

Information concerning the study, patent applications, processes, scientific data or other pertinent information is confidential and remains the property of the Sponsor. The investigator may use this information for the purposes of the study only.

It is understood by the investigator that the Sponsor will use information developed in this clinical study in connection with the development of the ablative or radiotherapy or surgical techniques and, therefore, may disclose it as required to other clinical investigators and to Regulatory Authorities. In order to allow the use of the information derived from this clinical study, the investigator understands that he/she has an obligation to provide complete test results and all data developed during this study to the Sponsor.

Verbal or written discussion of results prior to study completion and full reporting should only be undertaken with written consent from the Sponsor.

Therefore all information obtained as a result of the study will be regarded as CONFIDENTIAL, at least until appropriate analysis and review by the investigator(s) are completed.

Permission from the Executive/Writing Committee is necessary prior to disclosing any information relative to this study outside of the Trial Steering Committee. Any request by site investigators or other collaborators to access the study dataset must be formally reviewed by the TMG.

For peer review only

The results may be published or presented by the investigator(s), but the Funder will be given the opportunity to review and comment on any such results for up to 1 month before any presentations or publications are produced.

A Clinical Study Report summarising the study results will be prepared and submitted to the REC within a year of the end of study.

For peer review only

3.0 Informed Consent Form

ATLANTA

Additional **T**reatments to the **L**ocal tumour for metastatic prostate cancer: **A**ssessment of **N**ovel **T**reatment **A**lgorithms

INFORMED CONSENT FORM

Chief Investigator: Professor Hashim U. Ahmed

Principal Investigator: <<Insert Principal Investigator>>

Please initial each box below. Do not tick

1	I confirm that I have read and understand the patient information sheet dated _ _ / _ _ / _ _ _ _ (Version ___) for the **ATLANTA** Study. I have had the opportunity to consider the information, ask questions and have had these answered satisfactorily.	
2	I understand that the type of treatment I receive will be allocated using a randomisation process, and neither myself nor the staff involved in the study can influence this allocation.	
3	I understand that if at any point my medical condition changes, it may be necessary to withdraw from the trial and have treatment options reviewed. This will be discussed with me by clinicians and with my agreement.	
4	I understand that I may be asked questions relating to personal aspects such as about diet and lifestyle from my local research team.	
5	I understand that my participation is voluntary and that I am free to withdraw at any time, without giving reason and without my medical care or legal rights being affected.	
6	I understand that relevant sections of my medical notes and data collected during the study, may be looked at by individuals from the Sponsor of the trial (Imperial College London) and responsible persons authorised by the Sponsor, from regulatory authorities or from the NHS Trust, where it is relevant to my taking part in this research. I give permission for these individuals to have access to my records.	
7	I understand that the information collected about me will be used to support other research in future, and may be shared anonymously with other researchers.	
8	I give permission for all standard of care data and samples, including those taken prior to study recruitment, such as surgery specimen and biopsies including tissue, bloods, urine and imaging tests to be used in this study even if I withdraw at any points from the study	

For peer review only

9	I give permission for the researchers to contact me regarding this trial during this trial period.	
10	I agree for my GP and other doctors to be informed of my participation in this study and of any clinical relevant study results	
11	I agree to take part in the above study.	

All the boxes above must be initialled for consent to be valid

Please initial each box below. Do not tick

OPTIONAL		
12	I give permission for additional blood and urine samples to be taken and be made available for future research where the samples would be stored appropriately and the research approved separately ***(If you do not wish to give this permission, do not initial – you can still participate in the study).***	
13	I give permission for all standard of care samples and data such as surgery specimen, biopsies including tissue as well as imaging scans to be made available for future research where the samples and scans would be stored appropriately and the research approved separately ***(If you do not wish to give this permission, do not initial – you can still participate in the study).***	
14	I give permission for any blood, urine and tissue samples, which will look for changes in my genetic material (DNA) as described in the information sheet, to be used in this study. ***(If you do not wish to give this permission, do not initial – you can still participate in the study).***	
15	I give permission for any blood, urine and tissue samples, which will look for changes in my genetic material (DNA) as described in the information sheet, to be used for further ethically approved research in the field of prostate cancer research. ***(If you do not wish to give this permission, do not initial – you can still participate in the study).***	
16	I give permission for my samples and data from any scans to be sent and utilised in research both in the UK and worldwide. All material will be anonymous and I will not be identifiable. I understand that I will not be asked again for permission to run these additional research tests and I may also not be informed of the results ***(If you do not wish to give this permission, do not initial – you can still participate in the study).***	
17	I give permission for the researchers to contact me in the future regarding the possibility of further studies, but I understand that I am under no obligation to take part in these ***(If you do not wish to give this permission, do not initial – you can still participate in the study).***	
18	I give permission for all samples taken to be biobanked and transferred to the Imperial College Healthcare Tissue Bank (ICHTB) or other UK-based biobank for a period of up to 10 years (or per local policy) and will be used for histological, genomic and epigenetic analysis and for ethically approved future	

For peer review only

	studies by our team or other scientists interested in prostate cancer research. Samples will not have any personal information written on them. Researchers will not be able to identify you from your samples. ***(If you do not wish to give this permission, do not initial – you can still participate in the study).***	
19	I give permission for my name and NHS number to be used to obtain information about my health status from records held by the NHS and maintained by the NHS Information Centre and the NHS Central Register or any applicable NHS information system (including linkage to routine hospital admission data). I give this consent solely so that researchers may follow up on my health status for 10 years after my participation in the study. ***(If you do not wish to give this permission, do not initial – you can still participate in the study).***	
20	I give permission for my partial postcode to be recorded and stored. This will be used for research purposes only and will remain confidential. ***(If you do not wish to give this permission, do not initial – you can still participate in the study)***.	

Name of Participant Signature Date

____________________________ _____________________ _______________

Name of Person taking consent Signature Date

____________________________ _____________________ _______________

Please give one copy of the consent form to the patient, file one copy in the patient's medical records, and retain the original in the Investigator Site File

For peer review only

SPIRIT

STANDARD PROTOCOL ITEMS: RECOMMENDATIONS FOR INTERVENTIONAL TRIALS

SPIRIT 2013 Checklist: Recommended items to address in a clinical trial protocol and related documents*

Page No.	Section/item	Item No	Description
	**Administrative information**		
**1**	Title	1	Descriptive title identifying the study design, population, interventions, and, if applicable, trial acronym
**3**	Trial registration	2a	Trial identifier and registry name. If not yet registered, name of intended registry
**3**		2b	All items from the World Health Organization Trial Registration Data Set
**2**	Protocol version	3	Date and version identifier
**3,19**	Funding	4	Sources and types of financial, material, and other support
**1,2,19, 20**	Roles and responsibilities	5a	Names, affiliations, and roles of protocol contributors
**1, 20**		5b	Name and contact information for the trial sponsor
**19, 20**		5c	Role of study sponsor and funders, if any, in study design; collection, management, analysis, and interpretation of data; writing of the report; and the decision to submit the report for publication, including whether they will have ultimate authority over any of these activities
**19, 20**		5d	Composition, roles, and responsibilities of the coordinating centre, steering committee, endpoint adjudication committee, data management team, and other individuals or groups overseeing the trial, if applicable (see Item 21a for data monitoring committee)

For peer review only

	**Introduction**		
**5-10**	Background and rationale	6a	Description of research question and justification for undertaking the trial, including summary of relevant studies (published and unpublished) examining benefits and harms for each intervention
**5-10**		6b	Explanation for choice of comparators
**5**	Objectives	7	Specific objectives or hypotheses
**3, 10, 11, 12**	Trial design	8	Description of trial design including type of trial (eg, parallel group, crossover, factorial, single group), allocation ratio, and framework (eg, superiority, equivalence, noninferiority, exploratory)
	**Methods: Participants, interventions, and outcomes**		
**11**	Study setting	9	Description of study settings (eg, community clinic, academic hospital) and list of countries where data will be collected. Reference to where list of study sites can be obtained
**11**	Eligibility criteria	10	Inclusion and exclusion criteria for participants. If applicable, eligibility criteria for study centres and individuals who will perform the interventions (eg, surgeons, psychotherapists)
**5, 12-15**	Interventions	11a	Interventions for each group with sufficient detail to allow replication, including how and when they will be administered
**12-15**		11b	Criteria for discontinuing or modifying allocated interventions for a given trial participant (eg, drug dose change in response to harms, participant request, or improving/worsening disease)
**12-15**		11c	Strategies to improve adherence to intervention protocols, and any procedures for monitoring adherence (eg, drug tablet return, laboratory tests)
**12-15**		11d	Relevant concomitant care and interventions that are permitted or prohibited during the trial
**16**	Outcomes	12	Primary, secondary, and other outcomes, including the specific measurement variable (eg, systolic blood pressure), analysis metric (eg, change from baseline, final value, time to event), method of aggregation (eg, median, proportion), and time point for each outcome. Explanation of the clinical relevance of chosen efficacy and harm outcomes is strongly recommended

For peer review only - http://bmjopen.bmj.com/site/about/guidelines.xhtml

**Fig 1, Table 3**	Participant timeline	13	Time schedule of enrolment, interventions (including any run-ins and washouts), assessments, and visits for participants. A schematic diagram is highly recommended (see Figure)
**17-18**	Sample size	14	Estimated number of participants needed to achieve study objectives and how it was determined, including clinical and statistical assumptions supporting any sample size calculations
**17-18**	Recruitment	15	Strategies for achieving adequate participant enrolment to reach target sample size
	**Methods: Assignment of interventions (for controlled trials)**		
	Allocation:		
**12**	Sequence generation	16a	Method of generating the allocation sequence (eg, computer-generated random numbers), and list of any factors for stratification. To reduce predictability of a random sequence, details of any planned restriction (eg, blocking) should be provided in a separate document that is unavailable to those who enrol participants or assign interventions
**12**	Allocation concealment mechanism	16b	Mechanism of implementing the allocation sequence (eg, central telephone; sequentially numbered, opaque, sealed envelopes), describing any steps to conceal the sequence until interventions are assigned
**12**	Implementation	16c	Who will generate the allocation sequence, who will enrol participants, and who will assign participants to interventions
**N/A**	Blinding (masking)	17a	Who will be blinded after assignment to interventions (eg, trial participants, care providers, outcome assessors, data analysts), and how
**N/A**		17b	If blinded, circumstances under which unblinding is permissible, and procedure for revealing a participant's allocated intervention during the trial

	**Methods: Data collection, management, and analysis**		
**18**	Data collection methods	18a	Plans for assessment and collection of outcome, baseline, and other trial data, including any related processes to promote data quality (eg, duplicate measurements, training of assessors) and a description of study instruments (eg, questionnaires, laboratory tests) along with their reliability and validity, if known. Reference to where data collection forms can be found, if not in the protocol
**18**		18b	Plans to promote participant retention and complete follow-up, including list of any outcome data to be collected for participants who discontinue or deviate from intervention protocols
**18**	Data management	19	Plans for data entry, coding, security, and storage, including any related processes to promote data quality (eg, double data entry; range checks for data values). Reference to where details of data management procedures can be found, if not in the protocol
**17-18**	Statistical methods	20a	Statistical methods for analysing primary and secondary outcomes. Reference to where other details of the statistical analysis plan can be found, if not in the protocol
**17-18**		20b	Methods for any additional analyses (eg, subgroup and adjusted analyses)
**17-18**		20c	Definition of analysis population relating to protocol non-adherence (eg, as randomised analysis), and any statistical methods to handle missing data (eg, multiple imputation)
	**Methods: Monitoring**		
**18**	Data monitoring	21a	Composition of data monitoring committee (DMC); summary of its role and reporting structure; statement of whether it is independent from the sponsor and competing interests; and reference to where further details about its charter can be found, if not in the protocol. Alternatively, an explanation of why a DMC is not needed
**18**		21b	Description of any interim analyses and stopping guidelines, including who will have access to these interim results and make the final decision to terminate the trial

**18**	Harms	22	Plans for collecting, assessing, reporting, and managing solicited and spontaneously reported adverse events and other unintended effects of trial interventions or trial conduct
**18**	Auditing	23	Frequency and procedures for auditing trial conduct, if any, and whether the process will be independent from investigators and the sponsor
	**Ethics and dissemination**		
**18-19**	Research ethics approval	24	Plans for seeking research ethics committee/institutional review board (REC/IRB) approval
**18-19, 12, Suppl 2.0**	Protocol amendments	25	Plans for communicating important protocol modifications (eg, changes to eligibility criteria, outcomes, analyses) to relevant parties (eg, investigators, REC/IRBs, trial participants, trial registries, journals, regulators)
**12, Suppl 3.0**	Consent or assent	26a	Who will obtain informed consent or assent from potential trial participants or authorised surrogates, and how (see Item 32)
**12, Suppl 3.0**		26b	Additional consent provisions for collection and use of participant data and biological specimens in ancillary studies, if applicable
**17-18**	Confidentiality	27	How personal information about potential and enrolled participants will be collected, shared, and maintained in order to protect confidentiality before, during, and after the trial
**20**	Declaration of interests	28	Financial and other competing interests for principal investigators for the overall trial and each study site
**19, Suppl 2.0**	Access to data	29	Statement of who will have access to the final trial dataset, and disclosure of contractual agreements that limit such access for investigators
**Suppl 2.0**	Ancillary and post-trial care	30	Provisions, if any, for ancillary and post-trial care, and for compensation to those who suffer harm from trial participation

For peer review only

**19, Suppl 2.0**	Dissemination policy	31a	Plans for investigators and sponsor to communicate trial results to participants, healthcare professionals, the public, and other relevant groups (eg, via publication, reporting in results databases, or other data sharing arrangements), including any publication restrictions
		31b	Authorship eligibility guidelines and any intended use of professional writers
		31c	Plans, if any, for granting public access to the full protocol, participant-level dataset, and statistical code
	**Appendices**		
**Suppl 3.0**	Informed consent materials	32	Model consent form and other related documentation given to participants and authorised surrogates
**Suppl 3.0**	Biological specimens	33	Plans for collection, laboratory evaluation, and storage of biological specimens for genetic or molecular analysis in the current trial and for future use in ancillary studies, if applicable

*It is strongly recommended that this checklist be read in conjunction with the SPIRIT 2013 Explanation & Elaboration for important clarification on the items. Amendments to the protocol should be tracked and dated. The SPIRIT checklist is copyrighted by the SPIRIT Group under the Creative Commons "Attribution-NonCommercial-NoDerivs 3.0 Unported" license.

For peer review only
